# Molecular Dynamics Simulations and Classical Multidimensional Scaling Unveil New Metastable States in the Conformational Landscape of CDK2

**DOI:** 10.1371/journal.pone.0154066

**Published:** 2016-04-21

**Authors:** Pasquale Pisani, Fabiana Caporuscio, Luca Carlino, Giulio Rastelli

**Affiliations:** Department of Life Sciences, University of Modena and Reggio Emilia, Via Campi 103, 41125, Modena, Italy; Instituto de Tecnologica Química e Biológica, UNL, PORTUGAL

## Abstract

Protein kinases are key regulatory nodes in cellular networks and their function has been shown to be intimately coupled with their structural flexibility. However, understanding the key structural mechanisms of large conformational transitions remains a difficult task. CDK2 is a crucial regulator of cell cycle. Its activity is finely tuned by Cyclin E/A and the catalytic segment phosphorylation, whereas its deregulation occurs in many types of cancer. ATP competitive inhibitors have failed to be approved for clinical use due to toxicity issues raised by a lack of selectivity. However, in the last few years type III allosteric inhibitors have emerged as an alternative strategy to selectively modulate CDK2 activity. In this study we have investigated the conformational variability of CDK2. A low dimensional conformational landscape of CDK2 was modeled using classical multidimensional scaling on a set of 255 crystal structures. Microsecond-scale plain and accelerated MD simulations were used to populate this landscape by using an out-of-sample extension of multidimensional scaling. CDK2 was simulated in the apo-form and in complex with the allosteric inhibitor 8-anilino-1-napthalenesulfonic acid (ANS). The apo-CDK2 landscape analysis showed a conformational equilibrium between an Src-like inactive conformation and an active-like form. These two states are separated by different metastable states that share hybrid structural features with both forms of the kinase. In contrast, the CDK2/ANS complex landscape is compatible with a conformational selection picture where the binding of ANS in proximity of the αC helix causes a population shift toward the inactive conformation. Interestingly, the new metastable states could enlarge the pool of candidate structures for the development of selective allosteric CDK2 inhibitors. The method here presented should not be limited to the CDK2 case but could be used to systematically unmask similar mechanisms throughout the human kinome.

## Introduction

In eukaryotic organisms, phosphorylation is a common mechanism that regulates the activity of proteins involved in a large number of signaling pathways. The transfer of the γ-phosphate from ATP to a given protein substrate is catalyzed by protein kinases (PKs). These proteins constitute about 2% of all human genes and their tight regulation is responsible for the correct development and maintenance of eukaryotic organisms.[1,2] As a result of their pivotal roles, PKs are exposed to several layers of control that encompass allosteric effectors, post-translational modification, and alteration of sub-cellular localization.[2,3]

The fold of PKs is conserved throughout the whole family.[1] The naming convention based on the structure of the well-characterized cAMP dependent protein kinase (PKA) will be followed hereafter.[1,4] The fold is organized around a large hydrophobic helix (αF) and consists of a small N-terminal (N-lobe) and a larger C-terminal lobe (C-lobe). The N-lobe is formed by five antiparallel β-strands (β1-β5) coupled to the so-called αC-helix and contains two conserved embedded sequences: the Glycine-rich Loop and the AxK motif. The C-lobe has a high helical content (αD-αI) and contains four helices that compose the hydrophobic core (αD, αE, αF, and αH),[4,5] the PK catalytic machinery, including the so-called Catalytic Loop, and the highly conserved HRD and DFG motifs. The Asp of the DFG motif is responsible for the recognition of one of the ATP-bound Mg^2+^ ions. The Activation Loop (A-loop), which is positioned between the DFG and a third conserved motif, APE, is one of the most variable regions of PKs and is involved in substrate binding. The two lobes of PKs are connected by a unique short loop known as the “hinge region”. The phosphoryl transfer occurs in the deep cleft between the N and C-lobe. The relative positioning of the lobes influences the switch among the different conformational states. In particular, two conserved hydrophobic motifs, composed by non-consecutive residues and anchored to the αF-helix, are responsible for the correct positioning of the ATP molecule, the protein substrate, and the catalytic residues: the catalytic spine (C-spine), completed by the adenine ring of ATP, and the regulatory spine (R-spine), which is misaligned in PK inactive conformations.[3,4]

With regard to their function, PKs can be depicted as molecular switches that can exist in an “on” state, which is maximally active, and different inactive states. All PKs that have been crystallized in the active form share common features. The Lys residue of the AxK motif bridges to a conserved Glu in the αC-helix. This salt bridge stabilizes the α and β phosphate groups of ATP.[2] The A-loop adopts an extended conformation that allows the binding of the substrate and the catalysis, spine residues are correctly aligned and the key catalytic residues are in the correct position to bind ATP.[6–9] On the contrary, the crystal structures of inactive PKs show a greater conformational heterogeneity.[3,10] For example, the catalytic Asp of the DFG motif can adopt, at least, two distinct conformations: i) a DFG-in conformation, competent for the recognition and binding of ATP-bound Mg^2+^ ions; and ii) an inactive DFG-out conformation, in which the positions of Asp and Phe are flipped, resulting in an extended ATP binding pocket. Both DFG-out and DFG-in inactive conformations (also known as Src-like inactive conformations) have been largely investigated.[10–12] In the former the Lys-Glu salt bridge is maintained, whereas in the latter the αC-helix is displaced compared with the active state, the Lys-Glu salt bridge is lost, and the R-spine is disrupted. Moreover, in the Src-like inactive form, the A-loop is collapsed over the active site, thus blocking the access of both the nucleotide and the substrate.

PKs are targets of significant pharmaceutical interest. Inhibitors developed in the last decades can be classified according to their binding site.[13,14] Type I and I½ inhibitors compete with ATP for the nucleotide binding site. However, the high sequence and structural conservation of the ATP binding site hampers the achievement of drug selectivity, thus rising toxicity risks due to off-target effects.[15,16] For this reason, pharmaceutical research has focused on inhibitors that take advantage of extended or alternative binding sites only present in PK inactive forms, which show a larger conformational variability compared with the active forms. Type II inhibitors bind to the ATP binding site and extend to the adjacent hydrophobic site only accessible in DFG-out conformations.[17] Finally, true allosteric inhibitors bind in proximity of the αC helix (type III inhibitors) or to other cavities distal from the ATP binding pocket (type IV inhibitors).[13]

CDK2 is a serine/threonine kinase part of a larger group of kinases that have been first characterized as regulators of the eukaryotic cell cycle.[18] In association with the positive regulator cyclins E and A, CDK2 plays a pivotal role in the G1/S phase transition and drives the cell cycle through the S phase. The activity of CDK2 is also up-regulated by the phosphorylation of Thr160 in the A-loop. Deregulation of CDK2 has been implicated in several diseases, including different types of cancer.[19–21]

CDK2 has been crystallized in Src-like inactive, active (in association with cyclin and with both phosphorylated and unphosphorylated A-loop), and in DFG-out conformations.[7,22–24] In the Src-like form, the residues immediately following the DFG can form a short peculiar α-helical segment (αL12). Recently, structures of CDK2 in complex with the small fluorophore 8-anilino-1-naphtalenesulfonic acid (ANS) have been reported.[25] In these structures, two ANS molecules bind to an allosteric pocket formed by the αC-helix and the nearby β4-β5 strands, resulting in a large displacement of the αC-helix compared with all other CDK2 conformations. However, despite this large movement of the αC-helix, the positions of the C-spine, R-spine, and A-loop residues resemble those adopted in the Src-like inactive conformation.

Investigating the relative stability and the transitions among the different conformational states may provide great insights in the design of more selective PK inhibitors potentially able to exploit peculiar features of a given protein conformation. However, the study of structure/function relationships in such complex and dynamic systems requires a detailed molecular description.

In this context, the first issue is the representation of the associated conformational movements. Ideally, an exhaustive representation should be presented in a 3N-dimensional space, where N is the number of degrees of freedom of the system. In principle, it is always possible to represent the evolution of a system in a lower dimensional space that preserves the equilibrium thermodynamics properties. However, the construction of such spaces is not trivial and the coordinates are usually more or less a complicate function of the Cartesian coordinates with physical (*e*.*g*., energy) or geometric (*e*.*g*., distances, angles) meaning, or both. In this study, we propose to use the crystallographic data to build a low dimensional reference representation of a protein conformational space. In particular, we used the Root Mean Squared Deviation (RMSD) to assess differences among structures and classical Multidimensional Scaling (cMDS) to project the data in the low dimensional representation.

The second issue involves the timescale of the cellular events related to the activation/inactivation of PKs that is usually in the order of milliseconds.[26] Such scales can hardly be simulated even with modern computing resources. Traditionally, this problem has been addressed by using enhanced sampling techniques.[27] Examples reported in the literature include the study of the DFG flip or the closure of the A-loop using umbrella sampling and metadynamics, respectively.[28,29]

In the first part of the work, a large collection of CDK2 crystal structures was analyzed using cMDS. Crystal structures were projected in a two-dimensional conformational landscape and clustered showing that the low dimensional projection is able to maintain the correct separation among crystal structures of different conformational states. Moreover, key structural elements that distinguish the peculiar features of each cluster were identified. In the second part of the work, an out-of-sample extension of multidimensional scaling was used to analyze μs-scale plain and accelerated molecular dynamics simulations of CDK2 in the apo-form and in complex with ANS. Samples extracted from simulation trajectories were projected to the low-dimensional space obtained in the first part of the work, and crystal structure clusters were used as references to characterize the new samples. New metastable states that separate the active and inactive conformations of CDK2 were eventually found. In the final part of the work the potential of the presented protocol for drug discovery is discussed.

## Methods

### Crystal structure analysis

#### Dataset preparation

The Protein Data Bank database was filtered for CDK2 crystal structures annotated with the Uniprot ID P24941, *Homo sapiens* as source organism, and a resolution equal or better than 2.5 Å. This search returned 308 entries. The dataset was further filtered for structures where all residues chosen for the RMSD fit (see below) were unambiguously resolved. The final dataset was composed by 255 crystal structures.

#### Classical Multidimensional Scaling and Out-of-Sample Extension

Classical multidimensional scaling is a data analysis technique that allows a data dimensionality reduction i.e., mapping complex multidimensional data on a low-dimensional manifold. Differently from PCA, which operates on a covariance matrix, cMDS operates on a distance or dissimilarity matrix, as described below. Even if PCA and cMDS methods can return the same results in specific contexts, cMDS can be considered as a more general method that maintains its validity in a rigorous sense also for non-euclidean distances as RMSD, i.e., the metric chosen in this study.

In Euclidean spaces, by definition, distances between vectors are computed as:

$$\delta^{2}\left(u,v\right)={\left(u-v\right)}^{T}\left(u-v\right)=u^{T}u+v^{T}v-2\times \left(u^{T}v\right)$$ (1)

where ***u***^T^***v*** is the scalar product between ***u*** and ***v***. This formula is the basis for a relation between the inner product and Euclidean distances.

Given a set of *n* objects represented by *n* d-dimensional vectors ***x***_1_,…,***x***_*n*_*ϵ*ℜ^*d*^, the configuration matrix ***X*** = [***x***_***1***_]^T^…[***x***_***n***_]^T^ and the Gram matrix of the inner product between the vectors are defined as following:

$$B=XX^{T}$$ (2)

This is the basis to establish a connection between Euclidean distances and the inner product. An *n*×*n* pairwise dissimilarity matrix **Δ** = [*δ*_*ij*_], where *δ* is a function that measures the distance between the object *i* and *j*, can be converted in ***B*** through an operation known as “double centering”:

$$B=\tau \left(\Delta^{2}\right)=-\frac{1}{2}\left(I-\frac{m_{n}}{n}\right)\Delta^{2}\left(I-\frac{m_{n}}{n}\right)$$ (3)

where ***m***_*n*_ is an *n*×*n* matrix of masses for the *n* vectors (a matrix of ones if weights are all equals) and *τ* maps Euclidean squared distances to Euclidean inner products of an isometric configuration of points whose centroid is the origin.[30]

In general, the task of multidimensional scaling (MDS) can be stated as: given a matrix of generic pairwise distances **Δ** (including non-Euclidean distances), find a set of points ***x***_1_,…,***x***_*n*_*ϵ*ℜ^*p*^ embedded in a space (usually of lower dimensionality *p*) such that the new (Euclidean) distances ∥***x***_*i*_−***x***_*j*_∥ between them best preserve the original matrix **Δ**. In classical MDS a function called “strain”:

$$\text{Strain }\sim \;∥B-\langle x_{i},x_{j}\rangle {∥}^{2}$$ (4)

that represents the error in the reconstruction of centered scalar product is minimized instead of distances. The optimal solution in *p*-dimensional space is achieved by taking the *λ*_1_, …, *λ*_p_ non-zero eigenvalues of ***B*** with corresponding eigenvectors x*_k_ *= (x*_*1k*_, *x*_*1k*_, *…*, *x*_*1k*_*)*, *k = 1*, *…*, *p*, normalized by x*_k_*x*_*k*_ *= λ*_*k*_. Multidimensional scaling maintains a property of optimality in terms of ***B*** even when *δ* is not Euclidean and ***B*** = τ(**Δ**^**2**^) is not positive semidefinite with rank ≤ *d*.[31] In this case, ***B*** cannot be factorized to obtain a d-dimensional configuration of points.[30,32] This is circumvented by replacing ***B*** with ***B***^***’***^ the nearest (in terms of Frobenius norm) symmetric positive semidefinite matrix with rank ≤ *d*.[33]

The out of sample problem can be stated as the problem of projecting *m* new objects in the embedding ***B*** represented by the vectors {***x***_***1***_, …, ***x***_***n***_} found for the *n* reference samples. It is clear that calculating a new distance matrix **Δ**_***2***_ does not solve this problem, because ***B***_***2***_ (calculated on the new distance matrix) will represent a new embedding based on the *n* + *m* objects. In the work of Trosset and colleagues,[32] a solution for the simplest case of adding a single new point is given by defining:

$$A=\left[\begin{array}{cc} \Delta^{2} & a\\ a & 0 \end{array}\right]$$ (5)

where *a* is the vector of distances of the new point from the reference points and

$$B^{*}=\tau^{*}\left(\Delta^{2}\right)=\left[\begin{array}{cc} \tau \left(\Delta^{2}\right) & b\\ b^{t} & \beta \end{array}\right]$$ (6)

is obtained by applying the double centering on ***A*** but with a weight of zero for *a* (***m***_*n*_ term of Eq 3).

$${\text{min}}_{x\in {ℜ}^{d}}{\|B-\left[\begin{array}{c} \begin{array}{c} x_{1}^{t}\\ \vdots \end{array}\\ x_{n}^{t}\\ y^{t} \end{array}\right]\left[\begin{array}{cccc} x_{1} & \cdots & x_{t} & y \end{array}\right]\|}^{2}=\underset{x\in {ℜ}^{d}}{\text{min}}2\Sigma_{i=1}^{n}{\left(b_{i}-x_{i}^{t}y\right)}^{2}+{\left(\beta -y^{t}y\right)}^{2}$$ (7)

where *y* is the projection of the new point into the reference space. The global minimum of this function is the exact solution of the out-of-sample problem for cMDS but cannot be solved analytically. If the term *(β-y*^*t*^*y)*^*2*^ is dropped, then ***X***^*t*^***X*** is invertible and the unique solution is given by:

$$y\sim \hat{y}={\left(X^{t}X\right)}^{-1}X^{t}b$$ (8)

that represents and approximates the solution to the original problem (Eq 7).[32]

#### Dissimilarity calculation

The metric chosen to represent dissimilarity among structures and calculate Δ^2^ of Eq 3 is the Root Mean Square Deviation. An RMSD matrix among CDK2 crystal structures was calculated using the following residues according to the human CDK2 numbering: residues 4 to 12 (corresponding to β1 strand), 17 to 24 (β2), 29 to 34 (β3), 46 to 55 (αC), 66 to 71 (β4), 76 to 81 (β5), 87 to 93 (αD), 101 to 120 (αE), 121 to 135 (catalytic loop), 140 to 150 (including DFG), 182 to 194 (αF), and 277 to 282 (αI). This list of residues was characterized as the subset of residues that contains the largest part of the information about the structural variability of CDK2 and includes residues of R-spine (except for Met 196), C-spine, DFG, and the catalytic loop.

Samples were clustered using the hierarchical clustering method as implemented in SciPy 0.13.3.[34]

### Molecular dynamics simulations

#### Plain Molecular Dynamics

The coordinates of the different conformational states of CDK2 i) apo and ii) in complex with ANS were obtained from the crystal structure files deposited in the Protein Data Bank. The following PDB structures were used: 3PXF for the ternary complex between CDK2 and two molecules of ANS, 3PXR for the inactive closed state, and 1FIN for the active state.[7,25] 3PXR was chosen because it was crystallized by the same group that solved the crystal structure of 3PXF. A missing loop in the 3PXR crystal structure (Leu37-Glu40) was built with the homology modeling tools of the Schrödinger Suite 2014–3 by using as template the structure of the inactive CDK2 with PDB code 2EXM. The apo open state was obtained by removing the two molecules of ANS. The active state was obtained by removing Cyclin A and ATP from the 1FIN complex. The Cyclin/CDK2 complex was not included in this study because we were only interested in studying the apo-form of the enzyme.

ANS partial charges were calculated using the semi-empirical AM1-BCC charge model implemented in Antechamber.[35,36] Force field parameters were assigned using the ff14SB and GAFF force fields for the protein and ANS, respectively.[37,38] Hydrogens were added using the tleap tool implemented in Ambertools 14.[39]

The systems were solvated in truncated octahedron boxes with a 10 Å distance between the solutes and the edge of the periodic boxes. Long-range electrostatic interactions were treated by the Particle Mesh Ewald (PME) method with a 8 Å cutoff, and default values were selected for grid spacing and the order of B-spline for interpolation.[40] Molecular Dynamics simulations were performed using the cuda version of pmemd implemented in the AMBER 14 molecular dynamics package and a timestep of 2 fs.[39] Bonds involving hydrogens were restrained using the SHAKE algorithm.[41,42]

All systems were prepared for the production run using the following protocol:

1. A restrained minimization using 800 steps of the steepest descent method followed by 800 steps of the conjugate gradient method. The protein-ligand complex was submitted to three restrained-minimization steps: first the protein-ligand complex, then the protein, and, finally, ligand atoms were restrained.
2. An unrestrained minimization using 800 steps of the steepest descent method followed by 800 steps of the conjugate gradient method.
3. A thermalization phase in the NVT ensemble consisting in a first heating step from 0 K to 150 K in 150 ps, followed by a second heating step in which the temperature was increased from 150 K to 300 K in 150 ps while keeping the solute constrained.
4. A density equilibration step in the NPT ensemble consisting in six steps of 400 ps each with progressively reduced restraints on the solute.

The production runs of the plain MD were carried out in the NPT ensemble. The apo-system was simulated for 2 μs starting from the open conformation, for 1μs starting from the active conformation, and for 1μs starting from the Src-like inactive conformation (4 μs in total). The ternary complex was simulated for 1μs. Samples were collected every 40 ps for a total of 100,000 samples for the apo-system and 25,000 samples for the complex.

#### Accelerated MD

Accelerated MD simulations were performed on both systems using the “dual boost” method.[43] A boost potential was applied to all dihedral angles in the systems with input parameters E_dihed_ and α_dihed_. In addition, another boost potential was applied to all atoms in the systems with input parameter E_total_ and α_total_.

E_dihed_ was calculated as V_dihed_avg_+0.3*V_dihed_avg_ and α_dihed_ as 0.3*V_dihed_avg_/5, where V_dihed_avg_ is the average dihedral energy of the same system simulated with the plain MD.

E_total_ was calculated as V_total_avg_ + 0.2*N_atoms_ and α_total_ as 0.2*N_atoms_, where V_total_avg_ is the average potential energy of the respective plain MD simulation.

Samples were collected every 4 ps and the unbiased probability distributions were calculated using exponential averaging over histograms:

$$\text{P}\left(A_{i}\right)=P^{b}\left({\text{A}}_{i}\right)\frac{{\langle e^{\beta \Delta V\left(r\right)}\rangle }_{i}}{\sum_{i=1}^{M}{\langle e^{\beta \Delta V\left(r\right)}\rangle }_{i}},\;i=1,\ldots ,M$$ (9)

where P is the unbiased distribution, P^b^ is the biased distribution, <> represents averaging, ΔV is the boost factor of each sample and M is the number of bin.

The free energy was calculated as:

$$F=-k_{B}T\text{ln}P_{i}$$ (10)

### Simulation Analysis

Pairwise RMSD between structures and distance analysis were calculated using the library Prody 1.5.1.[44] Hydrogen bonds were identified and occupancy was calculated using a threshold of 3.5 Å and a D-H∙∙∙A angle larger than 120° with the program cpptraj.[45] π-π interactions were defined as an interaction between two aromatic rings in which either (a) the angle between the ring planes is less than 30° and the distance between the ring centroids is less than 4.4 Å (face-to-face), or (b) the angle between the ring planes is between 60° and 120° and the distance between the ring centroids is less than 5.5 Å (edge-to-face). Analysis of π-π interactions was implemented using the VMD 1.9.1 scripting language.[46]

All other calculations were implemented using Python 2.7, NumPy 1.8.2, and SciPy 0.13.3.[34,47,48]

## Results

### Crystal structure analysis

CDK2 is one of the most represented human PKs in the Protein Data Bank. The dataset of crystal structures used in this analysis was composed by 255 structures.

Relationships among crystal structures were modeled by calculating a dissimilarity matrix. Dissimilarity was measured as the RMSD between the backbone atoms of a subset of residues identified as the conserved core (see Methods). Then, cMDS was used to calculate a new set of coordinates associated to each structure. The new coordinates, which will be called here components,[31] were ranked according to the percentage of variance explained by each new coordinate. The first two components accounted for more than 80% of the variance and were used for the analyses discussed in the rest of the paper. The third and fourth components were visually inspected in association to the first two components and no further meaningful partition of the data associated to these components was found.

**Fig 1:**
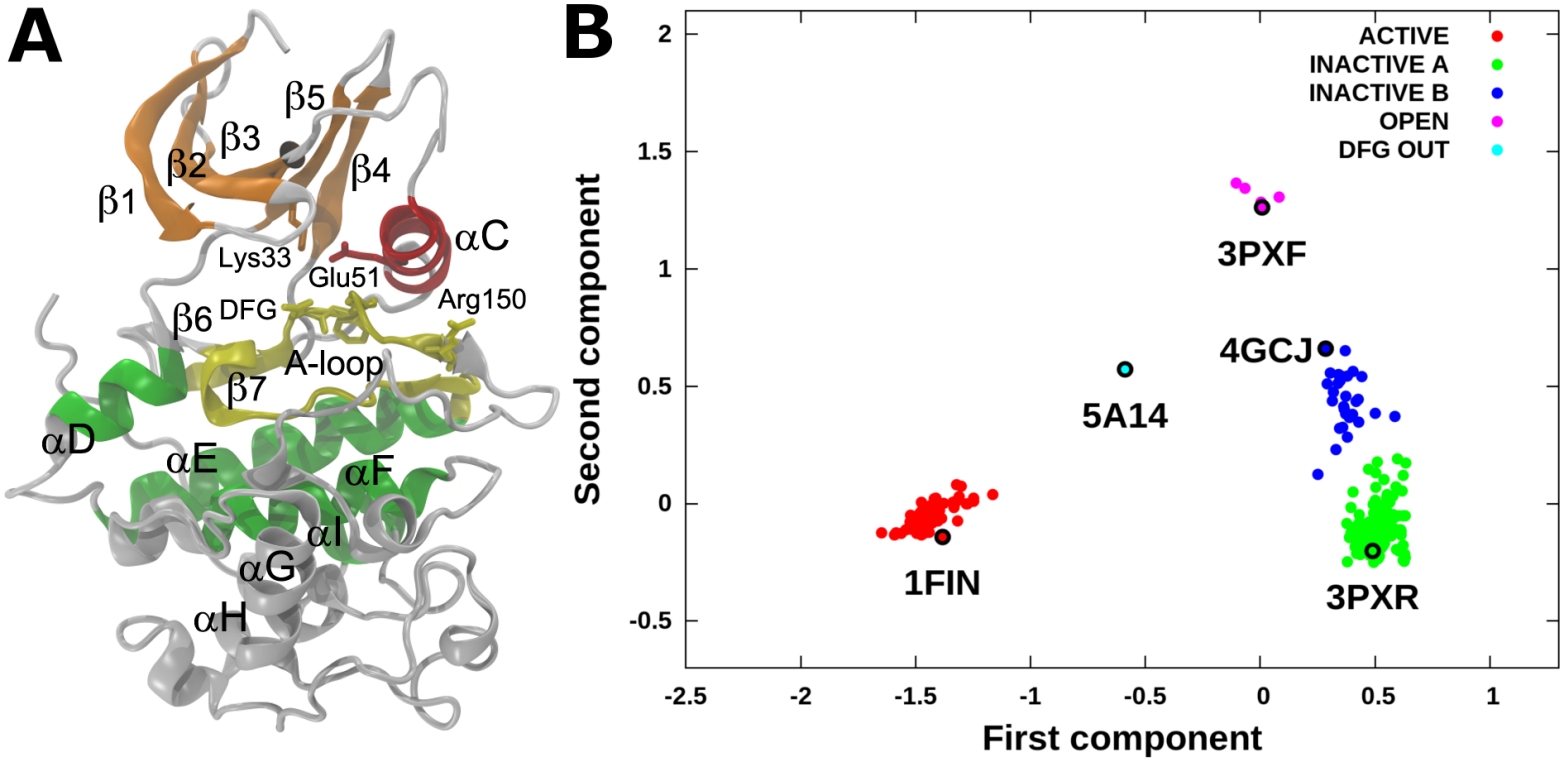
CDK2 conformational landscape. A) CDK2 structure in the active conformation (PDB code: 1FIN). Residues used for the calculation of the RMSD distance matrix are color coded: N-lobe β strands (orange), αC-helix (red), DFG and catalytic loop (yellow), and C-lobe helixes D-E-F-I (green). B) Conformational landscape calculated using 255 CDK2 crystal structures. Crystal structures were clustered in: active (red), type A inactive (green), type B inactive (blue), DFG-out (cyan), and open (pink). A representative for each cluster is reported.

As shown in Fig 1, the dataset was partitioned into five clusters using hierarchical clustering. The final results were robust regardless of the linkage method: the average, single, complete, and Ward method returned the same results. The K-means clustering method was discarded because it was not able to partition the DFG-out conformation as a separate cluster. In fact, partitioning the data using a number of clusters equal or larger than five with this method resulted in a not meaningful separation within inactive conformations, and the DFG-out structure was clustered among active or open conformations. This is probably due to the fact that the K-means method implicitly assumes gaussianity in the distribution of the samples.

**Fig 2:**
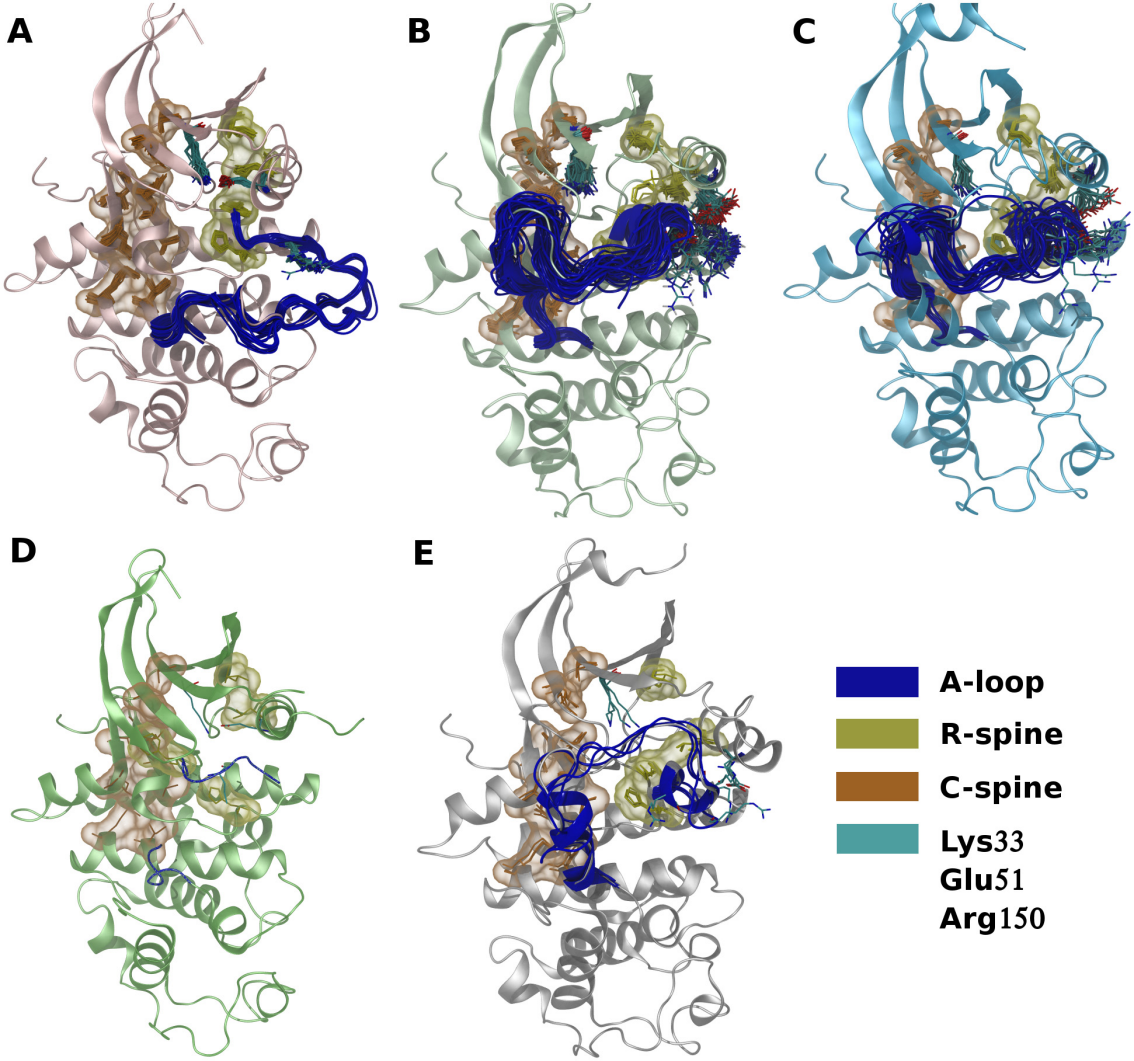
CDK2 crystal structure clusters. Crystal structures of each cluster superposed to a reference structure. The main structural elements are reported. A) Active (1FIN was used as reference); B) type A inactive (3PXR); C) type B inactive (4GCJ); D) DFG-out (5A14) and E) open (3PXF).

The main structural features of each cluster are shown in Fig 2. Throughout the text, we will refer to inactive CDK2 structures as *Src-like inactive* conformations, the structure with a flipped Phe146 as *DFG-out*, and the structures with an open ANS allosteric pocket as *open* conformations. The dataset was partitioned into 63 active, 186 Src-like inactive divided in two clusters of 156 (type A) and 30 (type B) structures, five open and one DFG-out structures. For each cluster a representative structure was chosen: 1FIN for active, 3PXR for type A inactive, 4GCJ for type B inactive, 3PXF for open, and 5A14 for DFG-out conformations.

In the cluster of active conformations (Fig 2A) i) the R- (Leu66, Leu55, Phe146, and His125) and C-spine (Ala31, Val18, Ile87, Leu133, Leu134, Ile135, Ile192, Met196) are correctly aligned; ii) the Lys33-Glu51 salt bridge is formed, and iii) the residues involved in the regulation and catalysis showed no significant deviation from the reference structure.

Src-like inactive conformations were split into two clusters: type A inactive (Fig 2B) and type B inactive (Fig 2C). The separation is mainly a consequence of the difference in the conformation of residues from 147 to 155 (the first part of the A-loop). This segment is folded into a short α helical structure (αL12) in type A inactive conformations, whereas it is unfolded in type B. As a consequence, Leu148 and Phe152 are differently positioned: in the type A cluster these two residues are located well inside the N-lobe near the C-spine, whereas in the type B one the side chains are shifted outward due to the differences in the backbone atoms (see S1 and S2 Figs). Interestingly, the side chains of these two residues point towards the hydrophobic pocket involved in the binding of ANS. It is important to note that the RMSD calculations include only residues from 147 to 150 (because the following residues in the A-loop are often not resolved), but the folding of the αL12 helix also causes remarkable differences in the backbone atoms of downstream residues 151–155. However, differences in the conformation of the residues included in our selection seem sufficient to account for the different conformation of the rest of the segment, as evidenced by the fact that type A and type B inactive structures were correctly separated. In both clusters the C-spine conformation is similar to that of active structures, whereas, both the R-spine and the Lys33-Glu51 salt bridge are broken. In fact, the C-helix is rotated compared with the active conformation, the side chain of Leu55 is moved away from the R-spine hydrophobic stack, and the side chain of Glu51 points outward making polar interactions with Arg150.

Open conformations form a separate cluster populated by the crystal structures of CDK2 in complex with the ANS molecules (Fig 1B). This is a consequence of the large shift of the αC-helix due to the presence of ANS in the allosteric pocket. The cluster, however, is very compact, in spite of the presence of apparently different conformations of the A-loop. As an example, 3PXF is characterized by a structured αL12, whereas in 3PXQ this segment is unfolded. However, 3PXF αL12 reveals distinctive structural features. The opening of the allosteric pocket, in fact, causes a shift in the relative positioning of the αC-helix, αL12, and the HRD motif compared with the inactive structures. As a consequence, Glu51 establishes a polar interaction with Arg126 (HRD motif) instead of Arg150 (αL12), which is too far (3PXQ, 3PY1, and 4EZ7) or completely rotated in the opposite direction (3PXF, 3PXZ). In all the open structures, Leu148 and Phe152 are shifted compared with type A inactive structures. In particular, Leu148 is shifted in order to accommodate the 8-aniline ring of the internal ANS and Phe152 is moved upward (3PY1) or completely away from the hydrophobic pocket (3PXF, 4EZ7). Despite differences in the fold of the αL12, the A-loop backbone atoms of the 151–155 segment of all open structures are more similar to those of type B than to those of type A inactive structures. All the above described structural features explain why the open cluster is closer to type B than to type A inactive structures in the conformational landscape. The Lys33, Glu51, C-spine, and R-spine positions are similar to their relative positions in both type A and type B inactive cluster members.

Finally, the DFG-out crystal structure (5A14) was projected halfway between active and inactive clusters in the cMDS landscape. This is in agreement with the fact that Glu51 is engaged in the salt bridge with Lys33 as in active conformations, thus stabilizing a more closed position of the αC-helix compared with Src-like inactive structures. The αL12 is unfolded, with the side chain of Leu148 and Phe152 oriented outward and the A-loop is disordered between residues 154 and 163.

### Molecular Dynamics simulations

Three different conformations of apo CDK2 (active, open, and Src-like inactive) and an open conformation of CDK2 in complex with two ANS molecules were simulated and analyzed through the use of the conformational landscape developed in the first part of the work and other order parameters reported in the literature.[49]

#### Apo CDK2 plain MD

Three apo CDK2 starting conformations were simulated: i) an active (1FIN); ii) an Src-like inactive (3PXR); and iii) an open (3PXF) conformation. Both the active and inactive conformations were simulated for 1 μs, whereas the open conformation simulation was extended to 2 μs, providing a total aggregated simulation time of 4 μs. The active and inactive systems were stable in all simulations, showing RMSD oscillations of 1.1±0.2 Å (active) and 1.2±0.1 (Src-like inactive) considering the residues used for cMDS or 2.4±0.2 Å (active) and 2.7±0.3 Å (Src-like inactive) considering the whole protein. The open system showed a larger deviation (1.8±0.3 Å considering cMDS residues or 3.1±0.3 Å considering the whole protein). RMSD were calculated from the starting PDB coordinates.

**Fig 3:**
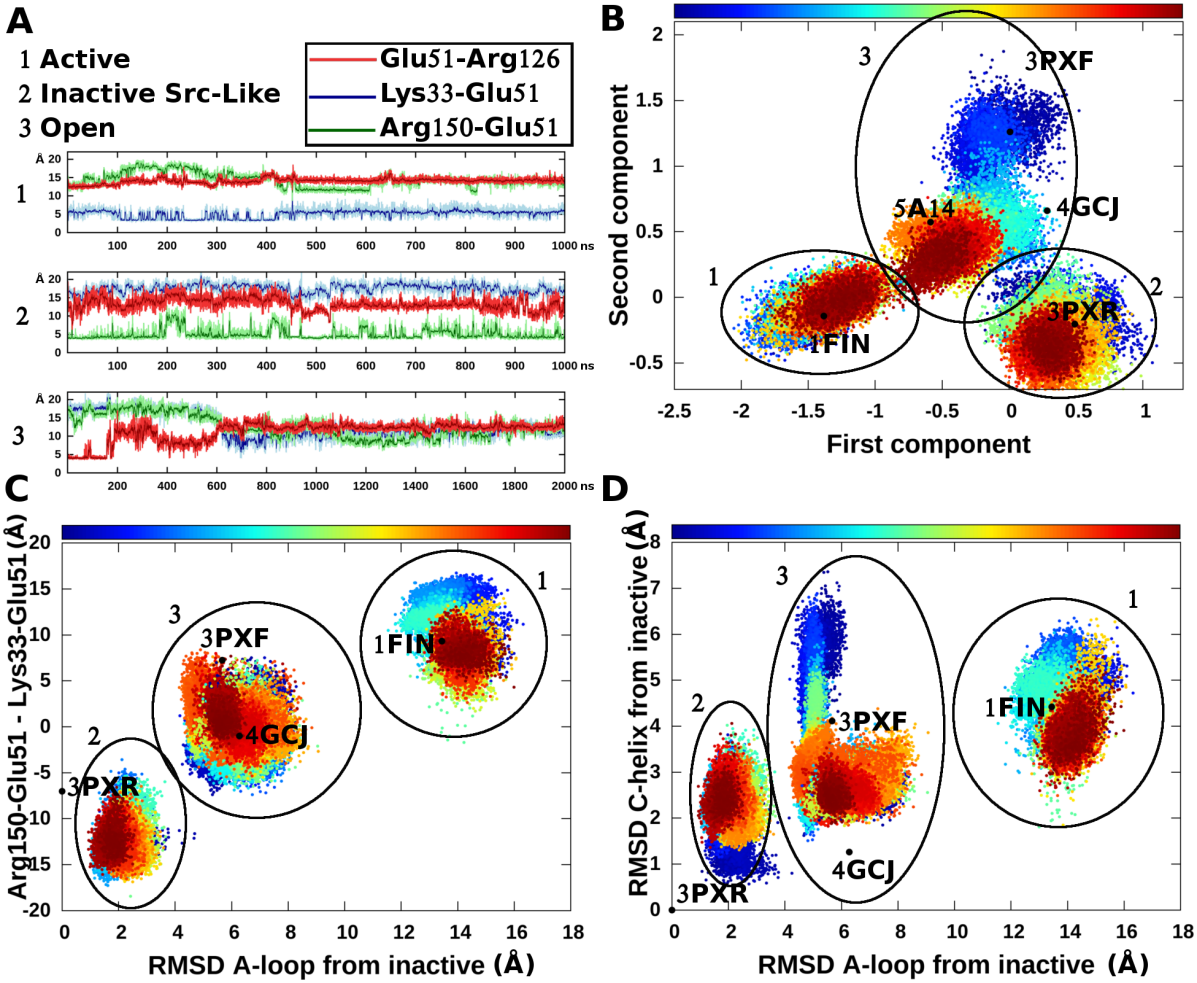
Apo CDK2 plain MD simulations. Plain MD simulations were numbered 1 (starting from the active conformation), 2 (from the inactive conformation) and 3 (from the open conformation). A) The distances between the donor-acceptor couples in Glu51-Arg126, Lys33-Glu51, and Arg150-Glu51 are reported for the three simulations B) Samples from these simulations were projected to the cMDS landscape and represented as a scatter plot. Snapshots were colored following the progress of the simulation (see the bars above the scatter plots). Colors run from blue at the beginning of the simulations to dark red at the end of the simulations. C) The same samples analyzed using the RMSD of the A-loop from the inactive conformation and the difference in distances between the couples Glu51-Arg150 and Lys33-Glu51. D) Representation of simulation samples using the RMSD of the A-loop and the RMSD of the αC-helix from the inactive conformation. Samples were colored from dark blue to dark red according to the simulation time.

Results from the simulations are summarized in Fig 3. The salt bridges formed by Glu51 in the active and inactive conformations were conserved throughout the simulation of these systems (Fig 3A). Glu51 forms a stable salt bridge with the side chain of Lys33 in the active state and with the side chain of Arg150 in the inactive one. The projection of trajectory samples to the previously calculated conformational landscape (Fig 3B) and alternative representations of the landscape obtained by using other relevant order parameters are shown in Fig 3C and 3D. According to the cMDS conformational landscape, in the simulations starting from the active and Src-like conformation the systems remain trapped within the conformational basin defined by the respective initial crystallographic poses (Fig 3B). This is in agreement with the representation of the conformational landscape obtained by using as order parameters: i) the difference in the Glu51-Arg150 and Glu51-Lys33 distances; ii) the RMSD of the A-loop; and iii) the RMSD of the αC-helix from the inactive state (Fig 3C and 3D). In order to define these last two measures 3PXR was used as reference.

On the contrary, the open structure drifts from the initial basin toward an intermediate state between the active and the Src-like inactive clusters (Fig 3B). Interestingly, the drift starts after ~600 ns, in which the system is quite stable, and is associated to the movement of the αC-helix toward the Src-like conformation and the partial closure of the allosteric site (see S3 Fig). In the first part of the simulation, the αC-helix swings with an RMSD between 2.0 and 7.0 Å from 3PXR (Fig 3D), whereas in the second part the αC-helix adopts a conformation similar to that of the Src-like inactive, with RMSD values between 1.5 and 3.5 Å (Fig 3D) as in the case of the simulation starting from the Src-like inactive conformation. Interestingly, in the open conformation Glu51 is initially involved in the formation of a salt-bridge with the side chain of Arg126 (HRD motif). However, this salt-bridge is disrupted at ~200 ns. The loss of this interaction may affect the stability of the open state and favor the closure of the helix. This movement is associated with the rotation of the Glu51 side-chain towards the interior of the N-lobe. Then, Glu51 rests in an intermediate position between the active and the Src-like inactive conformations without establishing the salt bridge with Lys33.

#### Apo CDK2 accelerated MD

**Fig 4:**
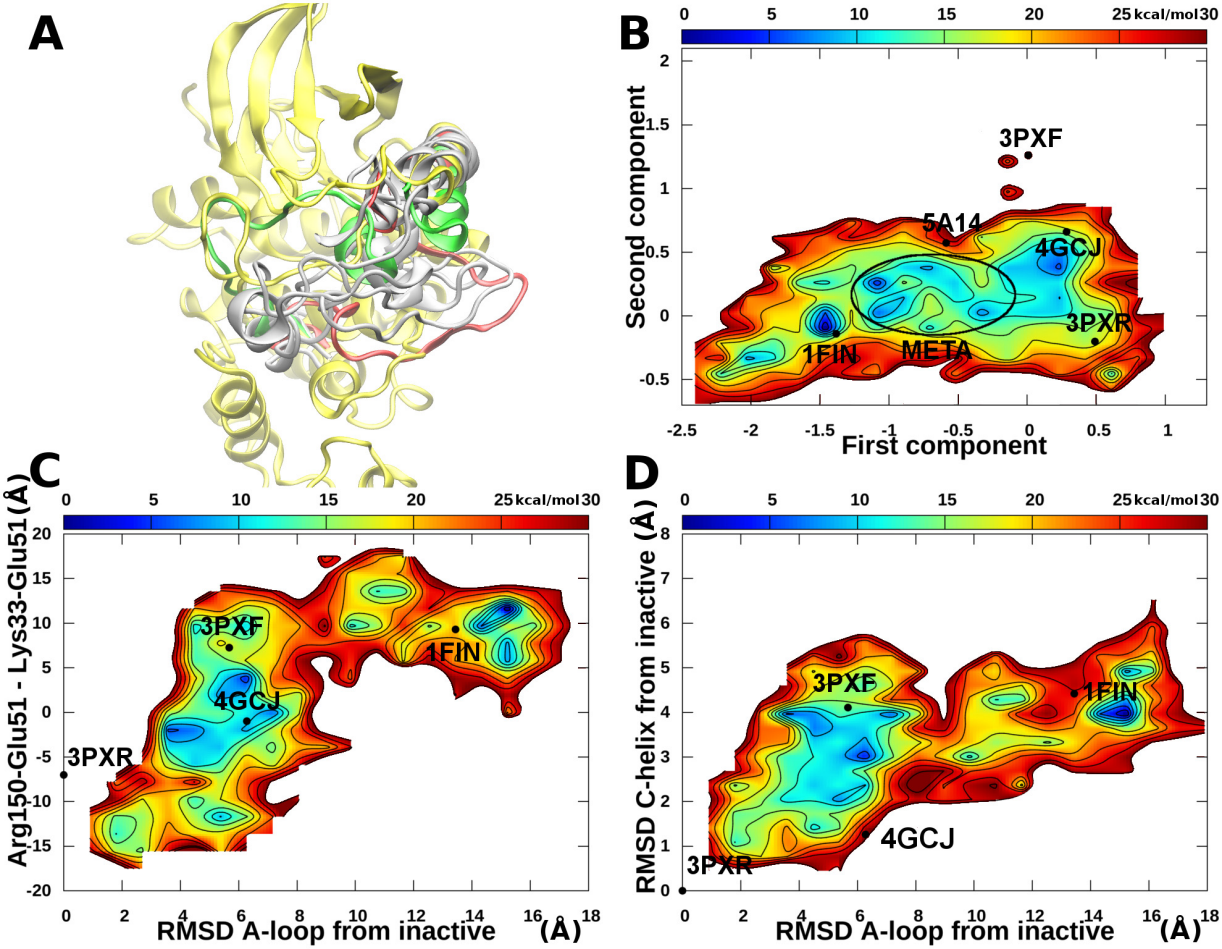
apo CDK2 accelerated MD simulations. A) The αC-helix and A-loop of metastable state representatives (white) and open (green) conformation superposed to the active conformation. B) Free energy landscape represented using the cMDS components. The metastable intermediate states are circled. C) Free energy landscape represented using the RMSD of the A-loop from the inactive conformation and the difference in distances between the couples Glu51-Arg150 and Lys33-Glu51. D) Free energy landscape represented using the RMSD of the A-loop and the RMSD of the αC-helix from the inactive conformation. Energies equal or above 30 kcal/mol from the global minimum were cut and represented in white for the sake of clarity.

Each of the previously described systems was also simulated with accelerated MD for 400 ns. Moreover, a random snapshot extracted from the plain MD of the open structure after the shift of the αC-helix and the rotation of Glu51 (Fig 3B, putative metastable intermediate state between the active and inactive ones) was also simulated for 400 ns. The sampling was further enhanced by simulating for 100 ns 10 random snapshots (with a uniform probability distribution) extracted from the plain MD of the open system. The final aggregated simulation time was 2.6 μs. Samples were reweighted using Eq 9 and free energies were calculated using Eq 10. Results are summarized in Fig 4, whereas the free energy landscapes of the individual simulations are shown in S4–S17 Figs.

A comparison of plain *vs* accelerated MD results revealed that in plain MD the different conformational basins are clearly disconnected, whereas with accelerated MD transitions among states can be observed and a more exhaustive sampling is achieved. In particular, the complete path among the open and closed states can be reconstructed for both the αC-helix and A-loop. During accelerated MD the formation and breaking of Glu51 salt bridges were also observed.

Interestingly, after reweighting, the active state is confirmed as a relevant free energy minimum (see Fig 4B and S4 Fig). This is an indication that the stability of this state observed during the plain MD was not an artifact due to the starting conformation and that apo CDK2 may populate this state even in the absence of cyclin. This is not the case of the open state, which assumes a very low weight and disappears as a minimum in the free energy landscape, thus suggesting that the presence of this state depends on the binding of the two ANS molecules. In fact, the open state is unstable and during accelerated MD evolves to the Src-like inactive basin (Fig 4B and S6 Fig) or to the metastable intermediate states as observed in the plain MD simulations. Representative structures of these states are reported in Fig 4A. Structures within the local minima circled in the landscape of Fig 4B are characterized by: i) a Glu51 side chain rotated towards the interior of the N-lobe without the formation of the Lys33-Glu51 salt bridge; ii) the side chains of Leu148 and Phe152 moved outside of the ANS pocket; and iii) a partially closed A-loop adopting different conformations. In the cMDS landscape, the metastable states are positioned in proximity of the DFG-out crystal structure (5A14), *i*.*e*., halfway between active and inactive conformations. However, this result should not come as a surprise because the DFG-out crystal structure and the metastable states share similar features in the conformation of the αC-helix and the first part of the A-loop (residues 147–152, *i*.*e*., the only part of the segment resolved in the DFG-out crystal structure). In any case, the DFG flip was never observed in our simulations, probably due to a high energetic barrier (Fig 4B). Free energy landscapes represented using different order parameters previously devised to study PKs[29,49,50] confirmed that the active and inactive states are indeed separated by metastable intermediates explored during our simulations (Fig 4C and 4D).

#### CDK2/ANS complex plain MD

**Fig 5:**
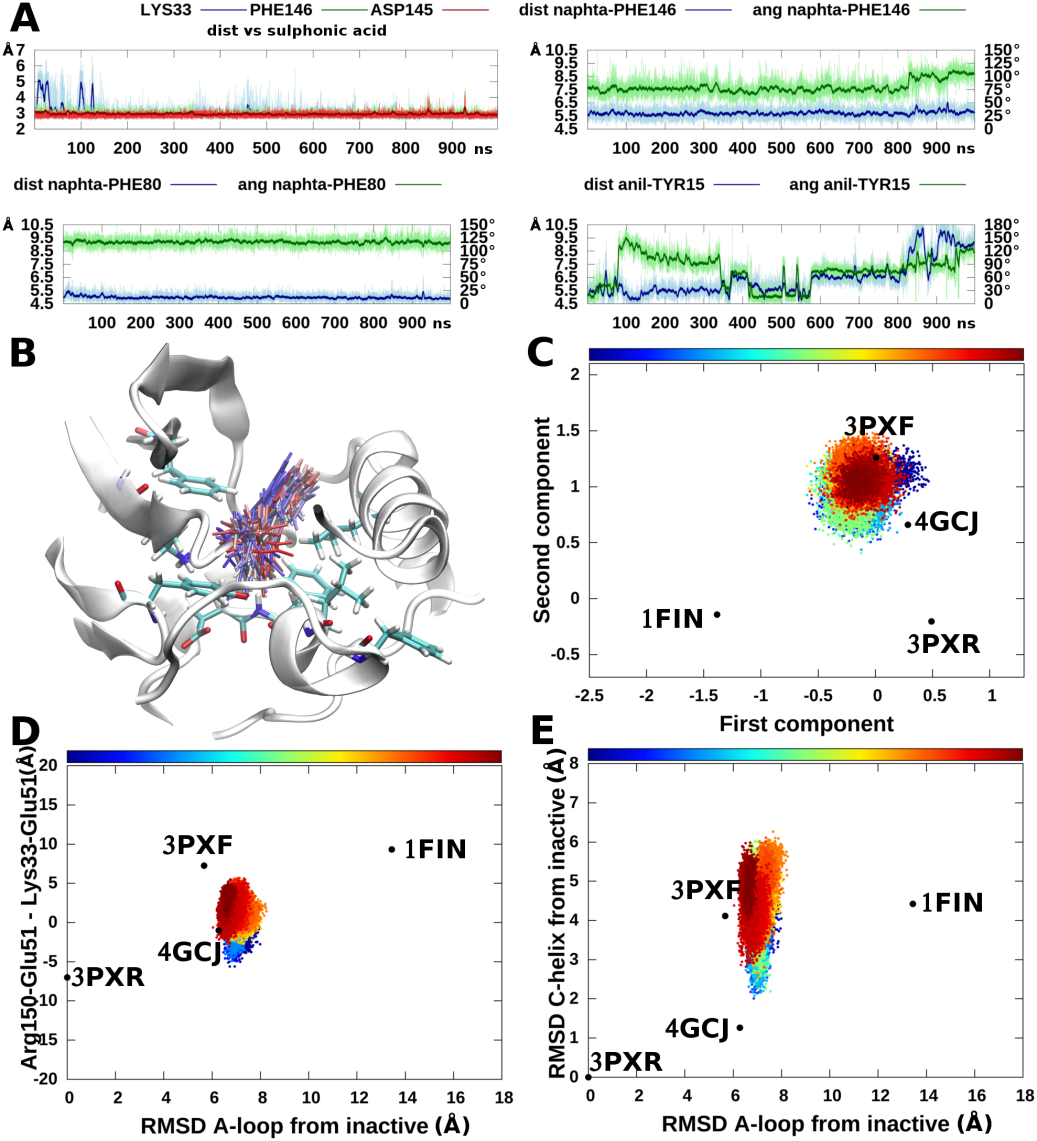
CDK2/ANS complex plain MD simulations. A) Binding interactions and B) Binding site of ANS1. C) Samples from plain MD were projected to the cMDS landscape and represented as a scatter plot. Snapshots were colored following the progress of the simulation (see the bars above the scatter plots). Colors run from blue at the beginning of the simulations to dark red at the end of the simulations. D) Representation of plain MD simulation samples using the RMSD of the A-loop from the inactive conformation and the difference in distances between the couples Glu51-Arg150 and Lys33-Glu51. E) Representation of plain MD simulation samples using the RMSD of the A-loop and the RMSD of the αC-helix from the inactive conformation.

The ternary complex of CDK2 and two ANS molecules was simulated for 1 μs. Throughout the simulation the CDK2 conformation was stable, with an average RMSD of 1.2±0.2 Å (residues used for cMDS) or 3.9±1.5 Å (whole protein) from the crystallographic structure. However, it is worthy of note that the two molecules of ANS (an internal, ANS1, and an external one, ANS2) showed a different behavior during the simulation. In particular, ANS1 showed very stable interactions with the protein and an average RMSD of 1.2±0.3 Å. On the contrary, ANS2 was very unstable (RMSD 4.7±1.6 Å) and did not maintain a well-defined pose. Results are summarized in Fig 5.

During the simulation the ANS1 molecule fits deeply inside the N-lobe and takes part to a rich network of hydrophobic interactions. The naphthalene moiety forms T-shape π-π interactions with the side chains of Phe80 and Phe146 (Fig 5A), and hydrophobic contacts with Leu55. A π-π interaction between Tyr15 and the ligand switches between a face-to-face and a T-shape interaction, but at ~500 ns the Tyr15 side-chain flips and the interaction is permanently lost. Polar interactions are mediated by the sulfonic acid moiety. In particular, ANS1 interacts with the Lys33 side-chain (30.2% occupancy) and the NHs of Asp145 (70.5%) and Phe146 (43.7%).

Throughout the simulation the αC-helix swings back and forth between an open and an Src-like inactive conformation showing RMSD values between 2.0 and 6.0 Å from 3PXR (Fig 5D). However, there is no appreciable drift toward the metastable or Src-like inactive conformations, in contrast to what observed for the simulation of the apo CDK2 starting from the open conformation. This may be related to the stability of the Glu51-Arg126 salt bridge, which is conserved for the whole simulation.

#### CDK2/ANS complex accelerated MD

**Fig 6:**
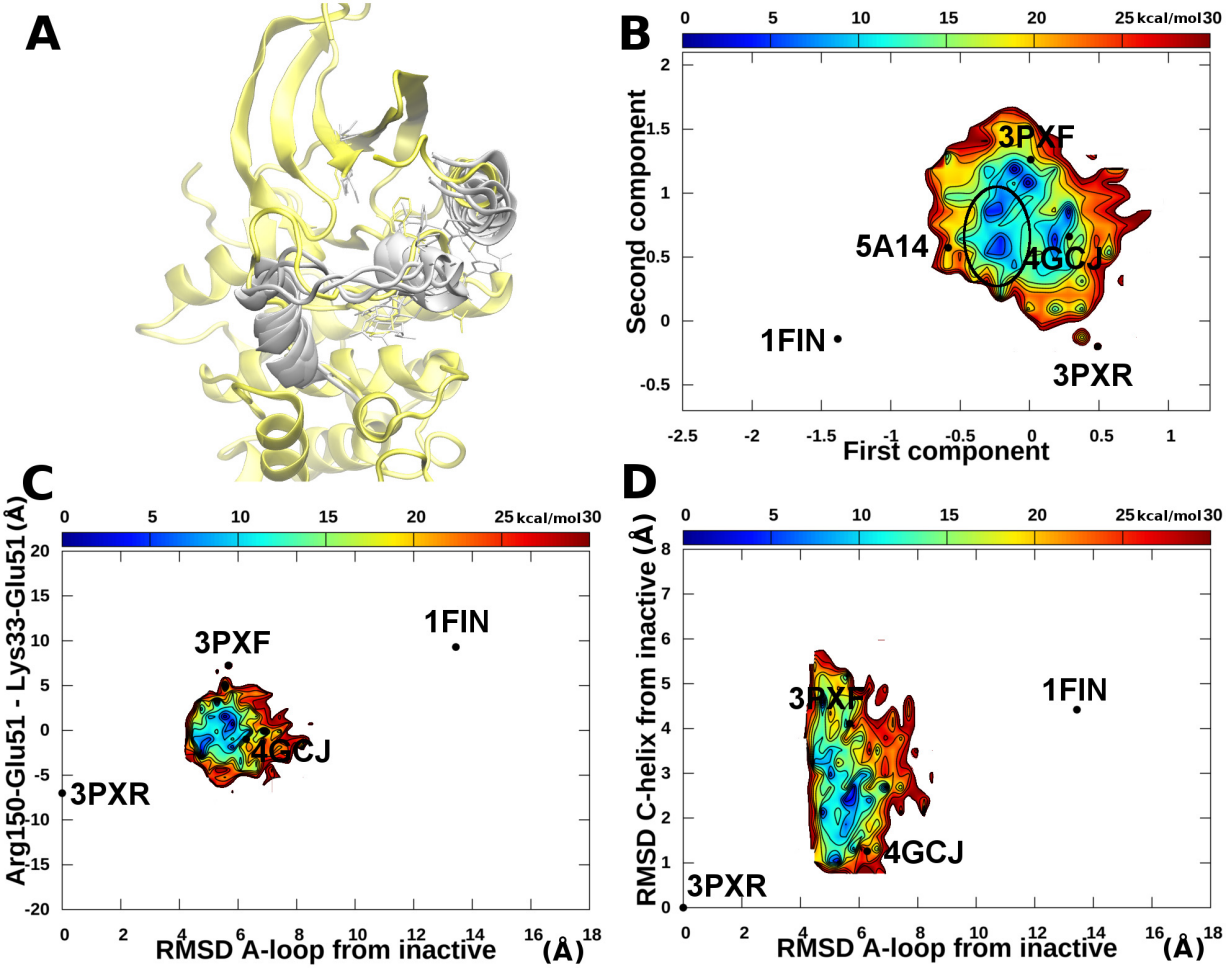
CDK2/ANS complex accelerated MD simulations. A) Representatives of free energy minima (white) superposed to the open conformation (yellow). B) Free energy landscape represented using the cMDS components. The free energy minima with flipped Leu148 and Phe152 are circled C) Free energy landscape represented using the RMSD of the A-loop from the inactive conformation and the difference in distances between the couples Glu51-Arg150 and Lys33-Glu51. D) Free energy landscape represented using the RMSD of the A-loop and the RMSD of the αC-helix from the inactive conformation. Energies equal or above 30 kcal/mol from the global minimum were cut and represented in white as the background for the sake of clarity.

The CDK2-ANS complex was also simulated for 400 ns using accelerated MD. A preliminary free energy calculation in the cMDS landscape revealed the presence of four putative free energy minima. Representatives from each minimum were randomly chosen and simulated for 400 ns each for a total aggregated simulation time of 2 μs. Representatives of the minima are reported in Fig 6A and the free energy landscapes are reported in Fig 6B–6D.

In the accelerated MD simulations the system was able to explore a larger range of conformations than in the plain MD ones. In agreement with the plain MD results, ANS1 maintained its interactions with the protein, whereas ANS2 did not show a well-defined binding mode, being involved in several events of binding and unbinding. Representatives of the two minima in proximity of 3PXF in the cMDS landscape (Fig 6B) showed structural features similar to the crystallographic complex, with the two molecules of ANS well accommodated within the allosteric pocket. The minima close to 4GCJ show features similar to the Src-like inactive structures, *i*.*e*., a closed αC-helix and the side chains of Phe148 and Leu152 pointing toward the ANS pocket. In our simulations, these conformations were explored after the loss of the Glu51-Arg126 salt bridge and the closure of the αC-helix.

Finally, two other minima (circled in Fig 6B) in the proximity of the metastable states observed in the apo simulations (circled in Fig 4B) were detected. In these structures the side chains of Leu148 and Phe152 point towards the exterior of the ANS pocket. Interestingly, in this case, the presence of ANS1 impedes the drift towards the active-like basin following the breaking of the Glu51-Arg126 salt bridge. In fact, the internal ANS prevents the rotation of the αC-helix to correctly position the Glu51 residue and the protein is forced to explore the inactive Src-like state.

## Discussion

### Building a Low Dimensional Conformational Landscape of CDK2

PKs may exist in different conformational states and transitions among these states consist in large rearrangements involving many degrees of freedom. Thus, describing such movements is not a trivial task. However, PKs are well-studied systems and a plethora of experimental structures corresponding to different states has been collected. In order to take advantage from this wealth of information we used crystal structures as reference points and devised a proper metric to calculate the similarity among them. Therefore, dissimilarities were used to construct a new space that best preserves the distinction among the reference points. Dissimilarities were stored in a matrix that is transformed in a new set of points by means of cMDS. The new points lie in a space in which the dimensions can be ranked by the amount of information explained, thus allowing to retain only the principal dimensions.

The accuracy of the landscape reconstruction was verified in two steps: in the first step the projected dataset was clustered and visually inspected; in the second step the new landscape was populated with an out-of-sample-extension of cMDS. In the out-of-sample embedding problem, *m* test objects are embedded in the representation space constructed from *n* training objects by cMDS. However, calculating a new distance matrix using the new points and applying cMDS to this matrix does not solve this problem, because the embedding of the original reference points would not be preserved.[32] The solution of the embedding problem implemented here is based on the work by Trosset and Priebe (see Methods, Eqs 5 and 6).[32] This solution has the advantage of being effective, fast and does not require the calculation of a new distance matrix among the new samples. According to the application proposed in our work, the number of new objects (generated by simulations) is usually much larger than the number of reference points (*m>>n*). Therefore, the asymptotic computational cost of calculating a new distance matrix would be O(*m*^2^), thus representing a possible issue for the analysis of very long simulations (or equivalently a high number of short simulations). On the other hand, the chosen solution requires only the calculation of distances from the reference points and scales linearly with the number of new samples.

In the case study reported in this paper, the differences among points in the reference dataset of CDK2 structures were calculated as the RMSD measured on a subset of atoms chosen as the “core” of the protein structure (see Methods), even if, in principle, other distances can be also used. RMSD has been previously chosen by Levy *et al*. as the distance measure to build the dissimilarity matrix.[51] As shown in Fig 1, in the new space the crystal structures were positioned and clustered consistently with known alternative CDK2 conformational states (*e*.*g*., active, inactive, DFG-out, open), indicating that the residues chosen to calculate dissimilarities are sufficient to account for the information needed to separate the conformational clusters. This can be better appreciated if the cMDS landscape is compared to the representation of the landscape obtained using other order parameters already reported in the literature to describe the conformational changes of PKs. Three previously used alternative structural order parameters were considered: i) the difference between the Arg150-Glu51 and Lys33-Glu51 distances; ii) the RMSD of the A-loop from the inactive conformation; iii) the RMSD of the αC-helix from the inactive state.[49] In the work by Pande and colleagues these order parameters were used to distinguish active and inactive conformations and identify intermediate states for the Src protein; however, we found that in CDK2 (as shown in Figs 3C, 4C, 5C and 6C) the open structures, the samples from metastable states and type B Src-like inactive (*e*.*g*., 4GCJ) could not be clearly distinguished using the first parameter. A better separation was obtained by taking into account the RMSD of the αC-helix (Fig 3D). However, the calculation of this class of order parameters has the drawback of defining a measure that depends on the choice of a single reference structure (in our work the crystal structure with code 3PXR was used as the reference conformation for the αC-helix and A-loop RMSD calculations). Moreover, different states may have the same RMSD value and collapse to a unique basin in the landscape representation. The same considerations arise for the calculation of the A-loop RMSD from a single reference structure. In this case, a second major drawback is due to the fact that in many crystal structures the A-loop is not resolved, and, therefore, dissimilarities cannot be calculated. On the contrary, a matrix of RMSD assures that the metric of the space is defined over multiple reference points, thus resulting in a finer and more robust representation.

### The Conformational Equilibrium of Apo CDK2

Given the timescales usually involved in conformational changes of PKs, a total aggregated time of 4 μs of plain MD may appear insufficient for a converged sampling of the entire path between active and inactive conformations. However, the analysis of plain MD results provided interesting information. In the plain MD starting from the active and Src-like inactive structures, the structural features of each conformation were conserved throughout the simulations (1 μs each). On the other hand, the simulation of the open conformation (with a displaced αC-helix and an open allosteric pocket) revealed interesting features that could not be inferred from the analysis of the crystal structures alone. In particular, the open CDK2 state did not appear to be stable if not in the presence of ANS, and the trajectories shifted toward newly identified metastable states with structural features in between the active and inactive structures (observed in both the plain and accelerated MD), or toward the type B inactive basin (observed in the accelerated MD), in both cases with the loss of polar interactions between Glu51 and Arg126. Both shifts are characterized by a more closed αC-helix and the consequent reduction of the size of the allosteric pocket compared with the starting conformation. The movement of Glu51 is also correlated to the position of Leu148 and Phe152. In fact, in order to penetrate into the N-lobe and establish the salt bridge with Lys33, Glu51 must leap over these two residues and Phe152 must flip outward. This sequence of movements is observed both in accelerated MD, where the formation of the Lys33-Glu51 salt bridge also occurs, and in the final part of the plain MD simulation starting from the open conformation. The analysis of the accelerated MD results confirmed that the main differences between open, Src-like inactive, and metastable basin samples depend on the αC-helix, the distance between Lys33 and Glu51 side chains, and the beginning of the A-loop. In the intermediate metastable states (circled in Fig 4B), the A-loop is still partially closed and remains partially closed also after the formation of the Lys33-Glu51 salt bridge (Fig 4B, free energy minimum near 1FIN).

### ANS Binding Causes a Shift in the Conformational Equilibrium of CDK2

The analysis of simulation of CDK2 in complex with two molecules of ANS returned a very different landscape, in which several basins could be observed. The first one (Fig 6) is populated by samples with an αC-helix conformation very similar to that of the 3PXF crystal structure, two bound ANS molecules, and the salt bridge between Glu51 and Arg126. On the other hand, the binding of the external ANS was characterized by several unbinding events. Moreover, in the cMDS landscape of the complex CDK2/ANS the other free energy minima correspond to conformations with an αC-helix similar to that of the Src-like inactive state (see Fig 6B). In these structures the hydrophobic pocket is still partially open because of the presence of ANS1 and the side chains of Leu148 and Phe152 point towards the interior (the two minima in proximity of 4GCJ) or the exterior (minima in proximity of the apo CDK2 metastable states) of the ANS pocket.

The salt bridge between Glu51 and Arg150 was never observed in the presence of ANS. In agreement with results previously reported for other PKs[49,52], our results suggest that the conformation of the first part of the A-loop is important for the switch of the Glu51 side chain from the inactive to the active position. In particular, when ANS is bound to CDK2, the negatively charged sulfonic acid of ANS1 replaces Glu51 in its interaction with Lys33, thus preventing the movement of the αC-helix toward the active position. We also suggest that, as in all free energy minima (including Src-like inactive conformations) the interaction of ANS1 are the same observed in the crystallographic complex, allosteric modulators could be also accommodated in conformations with a more closed αC-helix. Finally, the flexibility of the initial segment of the A-loop might provide alternative anchor points for the design of new small molecules that could bind to this pocket.

## Conclusions

A method to analyze PK conformational landscapes has been presented. The method allowed to efficiently analyze, cluster, and characterize a dataset of CDK2 crystal structures including structures in all known conformational states of this protein. The method was also extended to efficiently analyze a large number of samples generated by plain and accelerated MD.

CDK2 simulations allowed the exploration of the conformational landscape among active, open, and inactive crystal structures with the identification of new metastable conformations in between the well-known states.

Our results point toward a multistep process in which the inactive conformation is initially locked by polar interaction between Glu51 and Arg150 or Arg126. The loss of this polar interaction may allow the switch of Glu51 towards the interior of the N-lobe (required to form the Lys33-Glu51 salt bridge) and the concurrent closure of the αC-helix. In the intermediate metastable states, the A-loop remains partially closed. Interestingly, this is in agreement with the results reported for the intermediates previously observed in Hck[50] but in contrast to what observed for Src[49], in which an intermediate without the Lys-Glu ion pair (as in our case) but the A-loop completely unfolded was detected.

The application of the method to the CDK2 case allowed the identification of the peculiar features of the CDK2 conformation with an open allosteric pocket to be exploited for the design of new type III inhibitors. In particular, we showed that the internal ANS establishes stable interactions and its binding is crucial to shift the conformational equilibrium of CDK2 towards inactive conformations. On the contrary, the external ANS undergoes several binding and unbinding events. Moreover, our simulations suggested that ANS1 can be also accommodated in CDK2 conformations with an αC-helix similar to that of Src-like inactive structures. Finally, alternative configurations of the first part of the A-loop may influence the binding of type III inhibitors.

The generality of the method presented in this work allows its use to study the conformational variability of other PKs to unveil similar mechanisms throughout the kinome. Finally, the method can also be extended to the study of other protein families.

## Supporting Information

S1 Fig Crystal structures of the type A Src-like inactive conformation. The initial segment of the A-loop with the side chains of Lys33, Glu51, Arg126, Leu148, Arg150, and Phe152.
(TIFF)

S2 Fig Crystal structures of the type B Src-like inactive conformation. The initial segment of the A-loop with the side chain of Lys33, Glu51, Arg126, Leu148, Arg150, and Phe152.
(TIFF)

S3 Fig Apo CDK2 plain MD metastable conformation. Some features of the metastable conformation at the end of the apo CDK2 plain MD are reported. The initial structure (3PXF) is reported in white as reference. The αC-helix of the metastable conformation is more closed (red), Glu51 is inside the N-lobe (red, in stick), the A-loop is still closed but the side chain of Phe152 is flipped outside (yellow).
(TIFF)

S4 Fig Free energy landscape derived from the accelerated MD simulation of the active conformation. (TIF)

S5 Fig Free energy landscape derived from the accelerated MD simulation of the inactive conformation. (TIF)

S6 Fig Free energy landscape derived from the accelerated MD simulation of the open conformation. (TIF)

S7 Fig Free energy landscape derived from the accelerated MD simulation of a representative of the metastable intermediate state. (TIF)

S8 Fig Free energy landscape derived from the accelerated MD simulation of a random snapshot extracted from the plain MD of the open system. (TIF)

S9 Fig Free energy landscape derived from the accelerated MD simulation of a random snapshot extracted from the plain MD of the open system. (TIF)

S10 Fig Free energy landscape derived from the accelerated MD simulation of a random snapshot extracted from the plain MD of the open system. (TIF)

S11 Fig Free energy landscape derived from the accelerated MD simulation of a random snapshot extracted from the plain MD of the open system. (TIF)

S12 Fig Free energy landscape derived from the accelerated MD simulation of a random snapshot extracted from the plain MD of the open system. (TIF)

S13 Fig Free energy landscape derived from the accelerated MD simulation of a random snapshot extracted from the plain MD of the open system. (TIF)

S14 Fig Free energy landscape derived from the accelerated MD simulation of a random snapshot extracted from the plain MD of the open system. (TIF)

S15 Fig Free energy landscape derived from the accelerated MD simulation of a random snapshot extracted from the plain MD of the open system. (TIF)

S16 Fig Free energy landscape derived from the accelerated MD simulation of a random snapshot extracted from the plain MD of the open system. (TIF)

S17 Fig Free energy landscape derived from the accelerated MD simulation of a random snapshot extracted from the plain MD of the open system. (TIF)
