# Supplementary figures and images for: Molecular Dynamics Simulations and Classical Multidimensional Scaling Unveil New Metastable States in the Conformational Landscape of CDK2
Source: PLoS One. 2016 Apr 21;11(4):e0154066. doi: 10.1371/journal.pone.0154066 (PMC4839568; doi:10.1371/journal.pone.0154066)

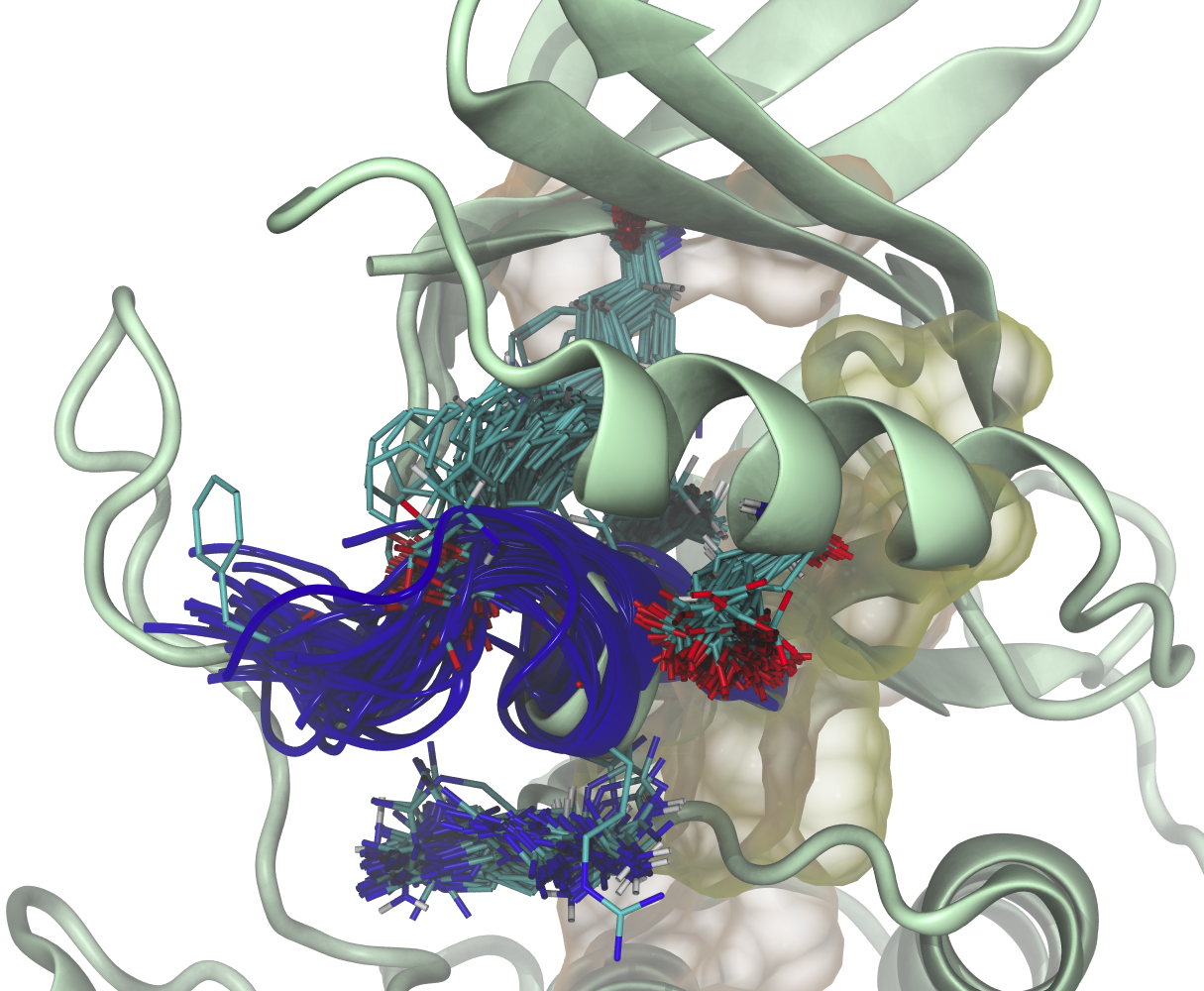

Supplement: S1 Fig — The initial segment of the A-loop with the side chains of Lys33, Glu51, Arg126, Leu148, Arg150, and Phe152. (TIFF) [file pone.0154066.s001.tiff]

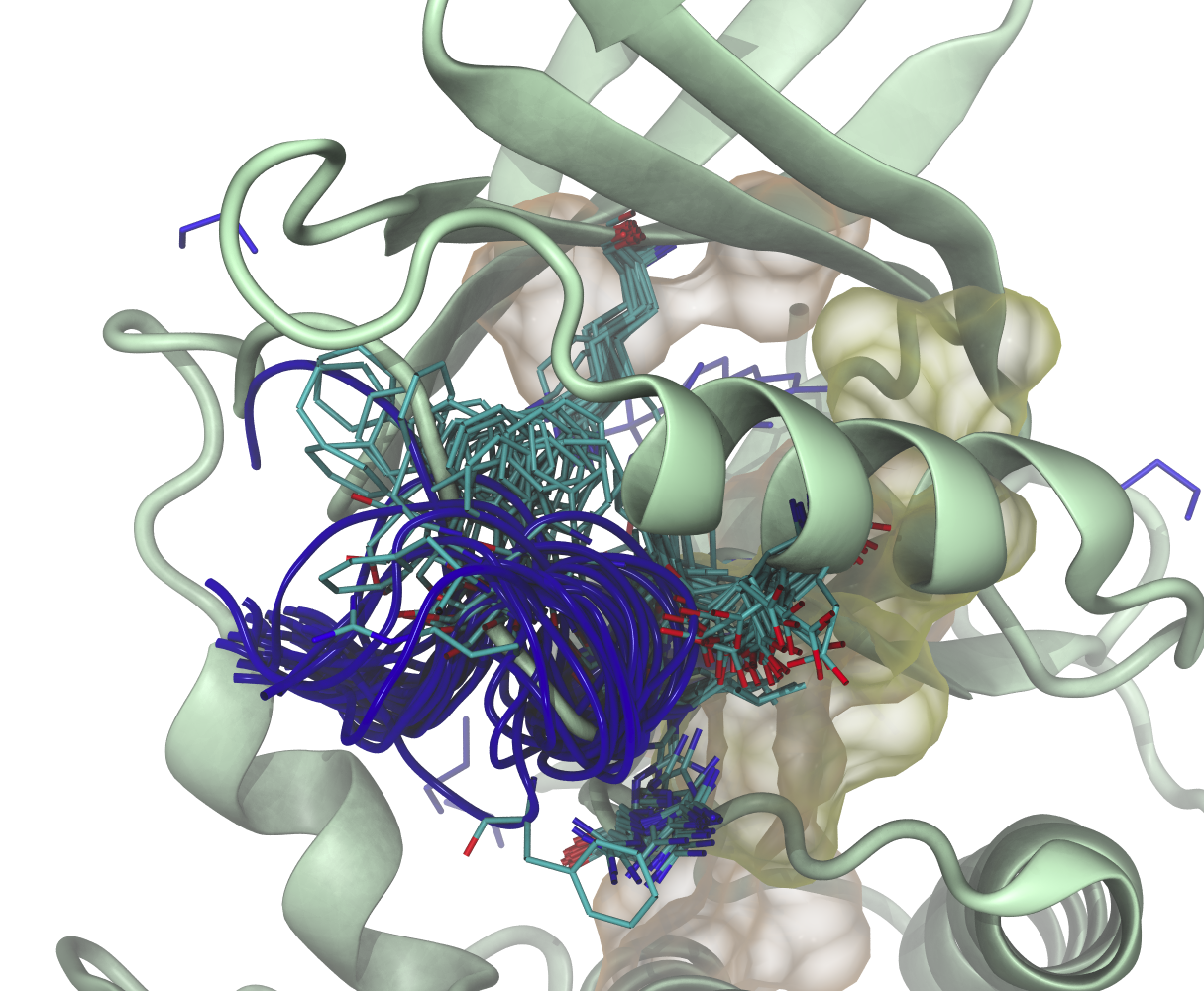

Supplement: S2 Fig — The initial segment of the A-loop with the side chain of Lys33, Glu51, Arg126, Leu148, Arg150, and Phe152. (TIFF) [file pone.0154066.s002.tiff]

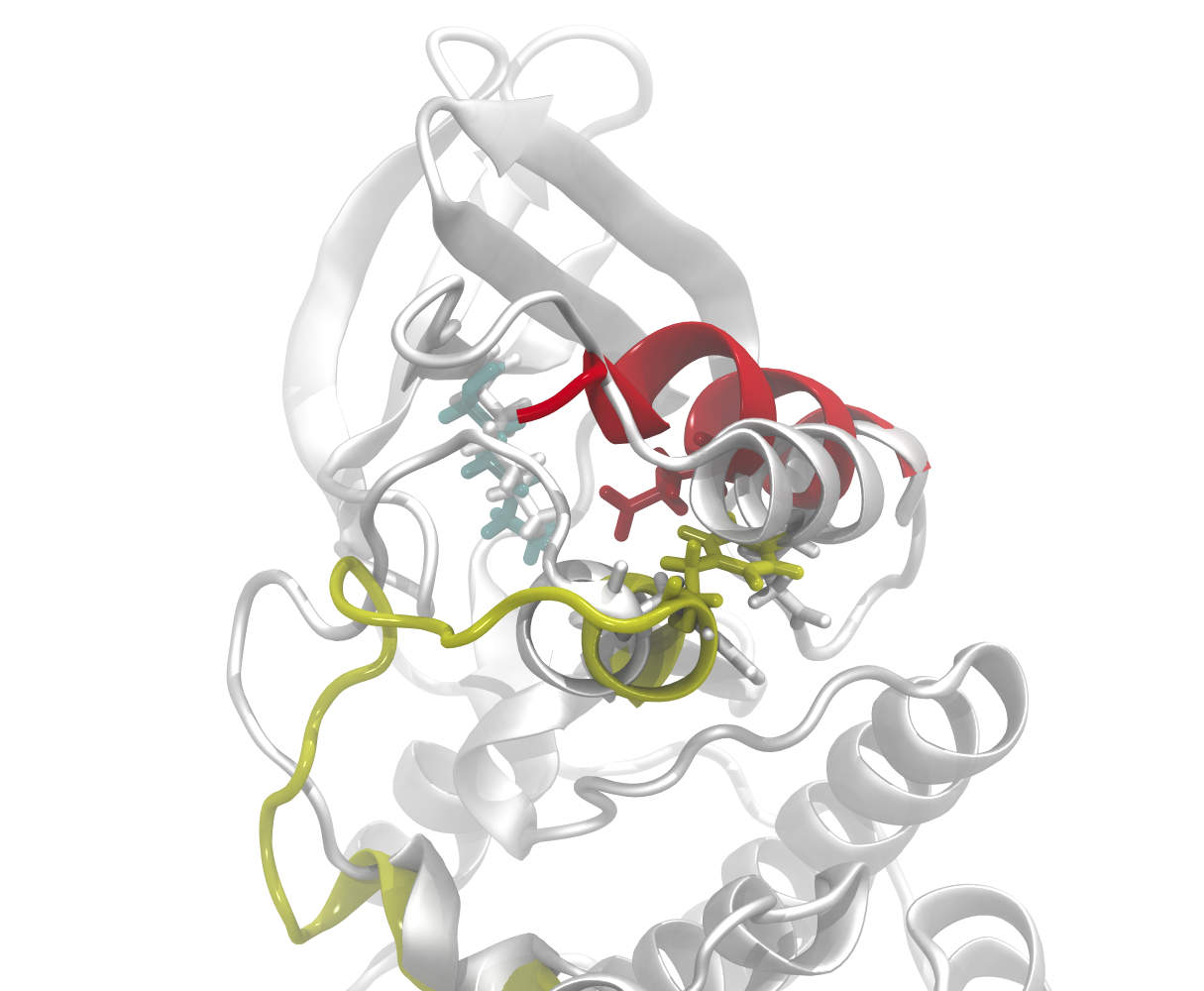

Supplement: S3 Fig — Some features of the metastable conformation at the end of the apo CDK2 plain MD are reported. The initial structure (3PXF) is reported in white as reference. The αC-helix of the metastable conformation is more closed (red), Glu51 is inside the N-lobe (red, in stick), the A-loop is still closed but the side chain of Phe152 is flipped outside (yellow). (TIFF) [file pone.0154066.s003.tiff]

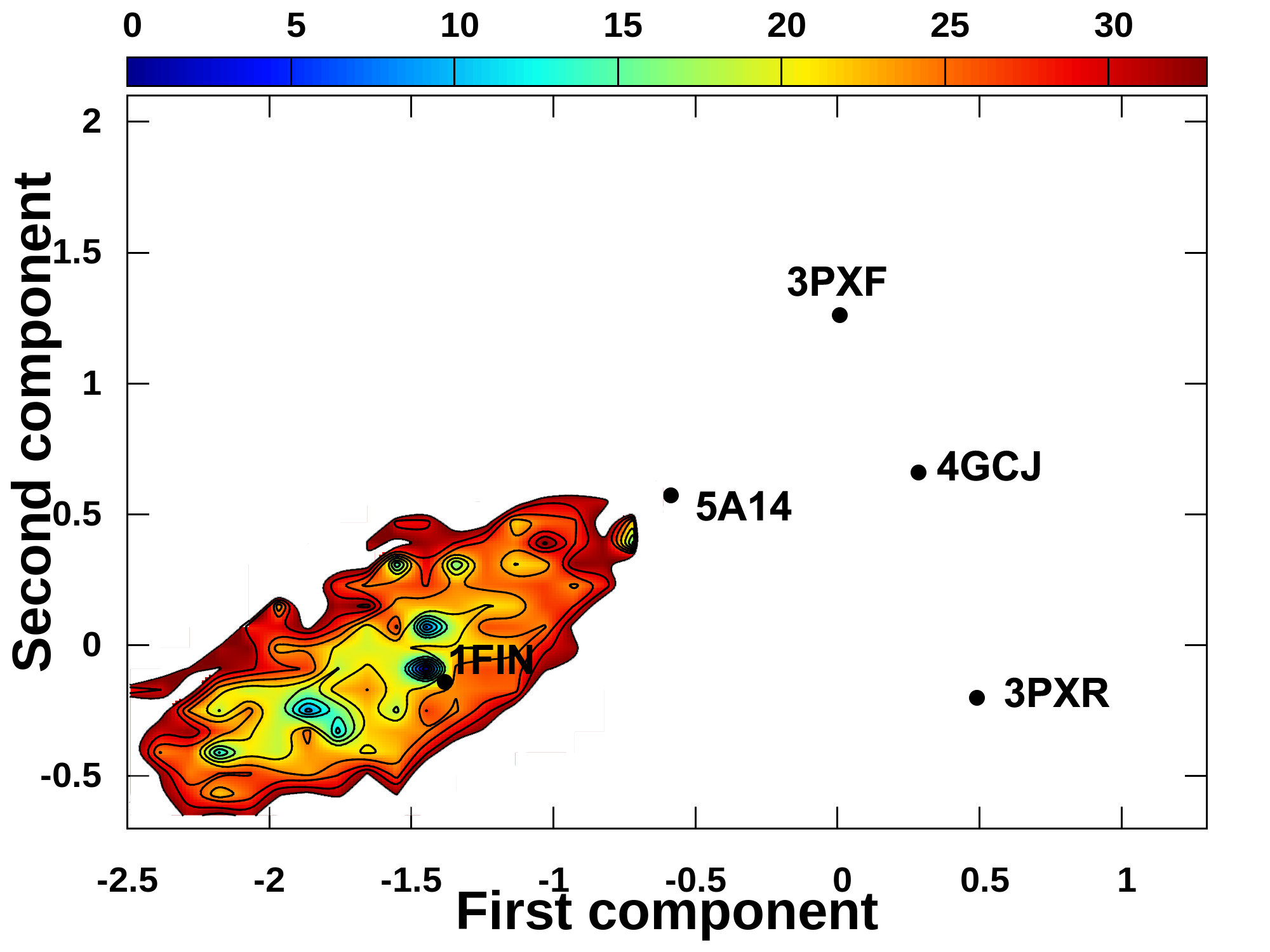

Supplement: S4 Fig — (TIF) [file pone.0154066.s004.tif]

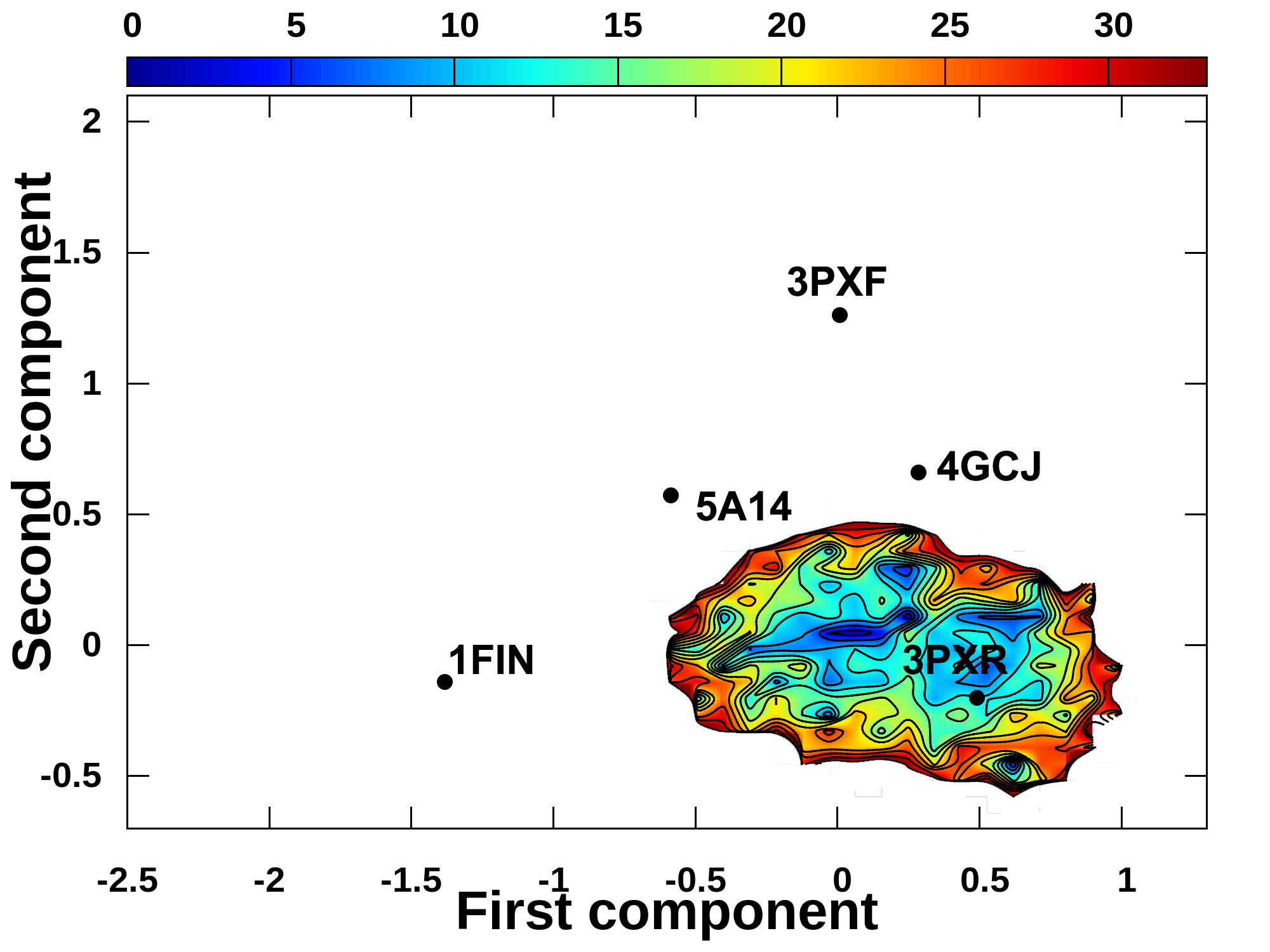

Supplement: S5 Fig — (TIF) [file pone.0154066.s005.tif]

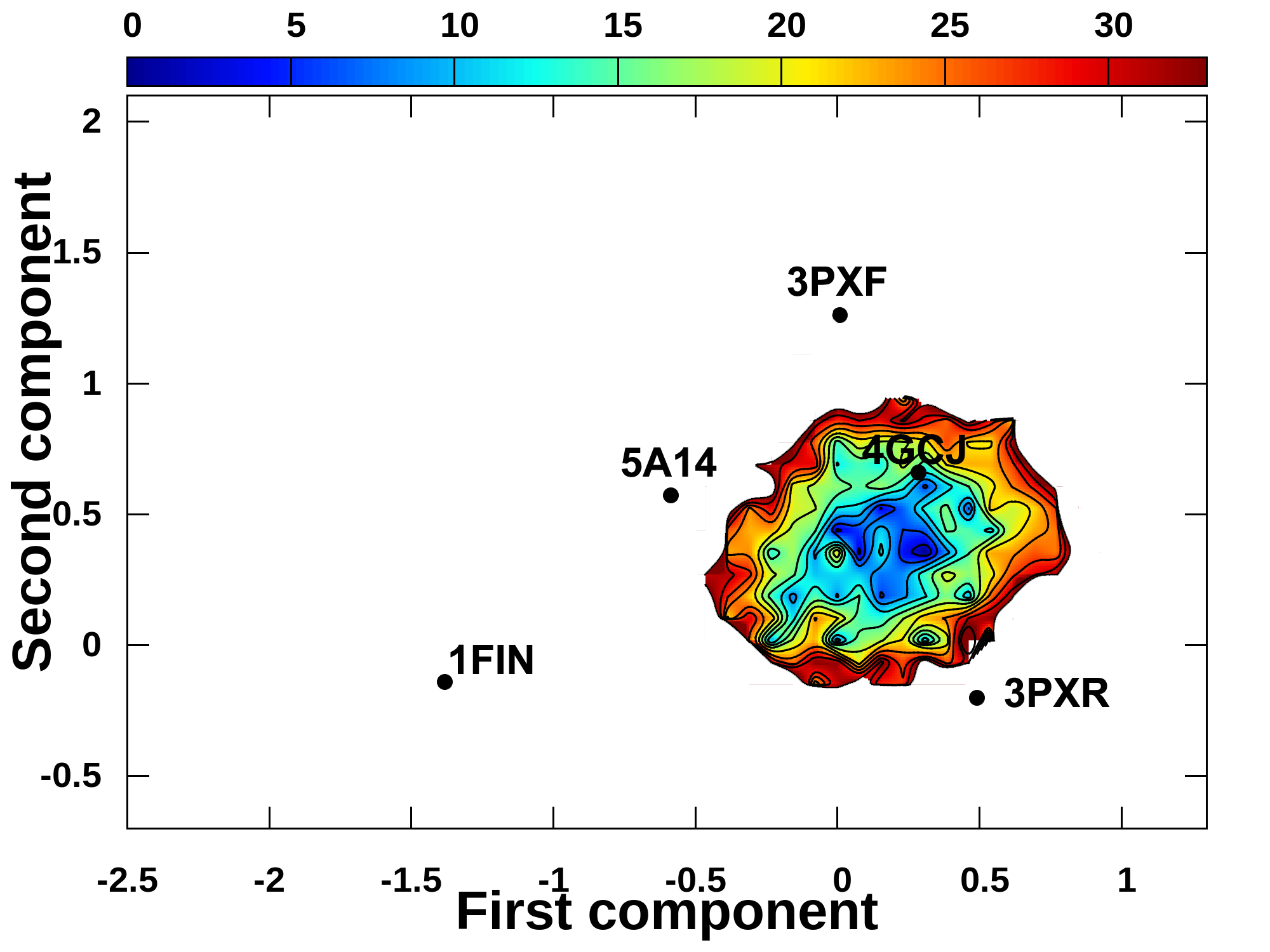

Supplement: S6 Fig — (TIF) [file pone.0154066.s006.tif]

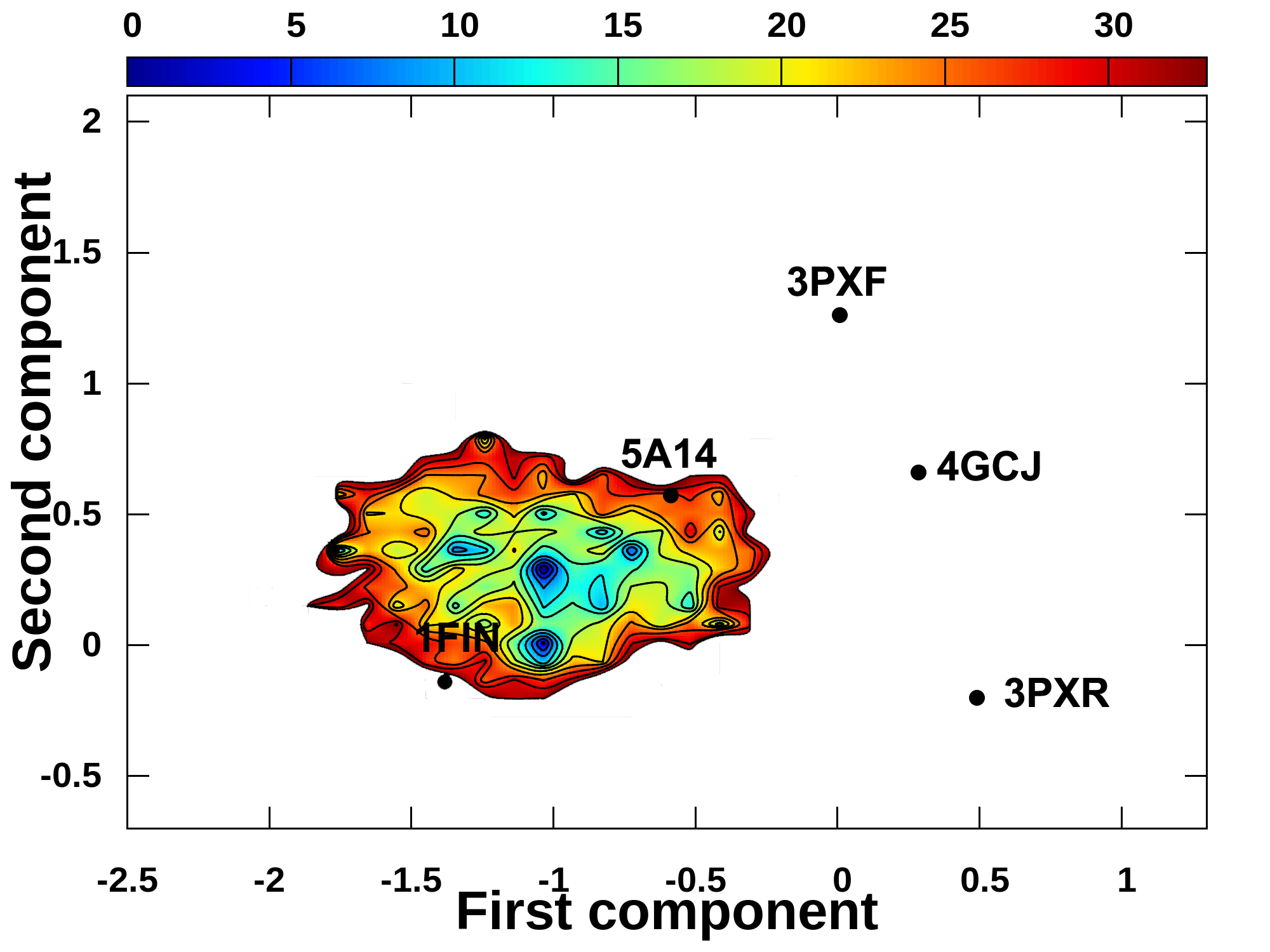

Supplement: S7 Fig — (TIF) [file pone.0154066.s007.tif]

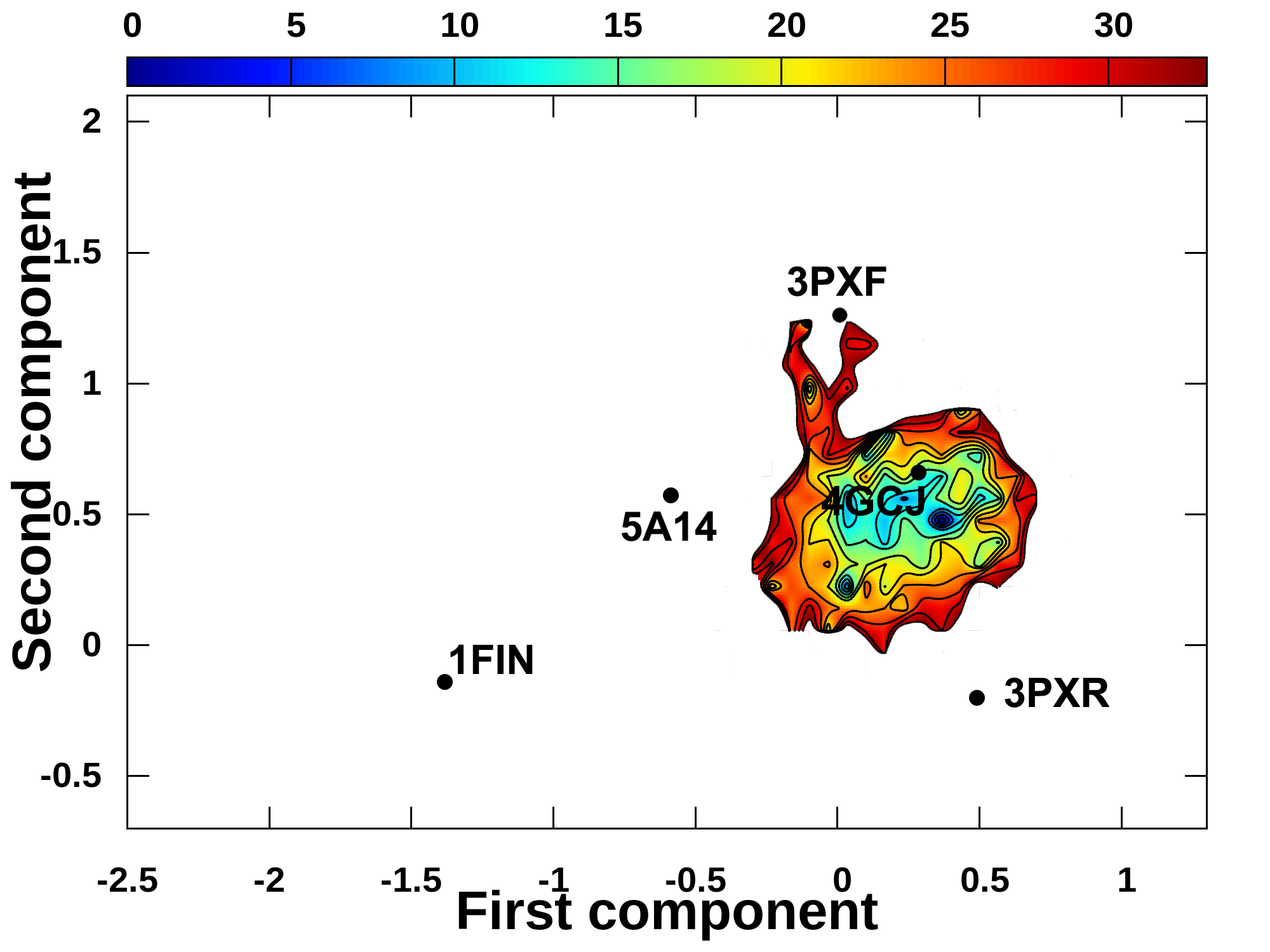

Supplement: S8 Fig — (TIF) [file pone.0154066.s008.tif]

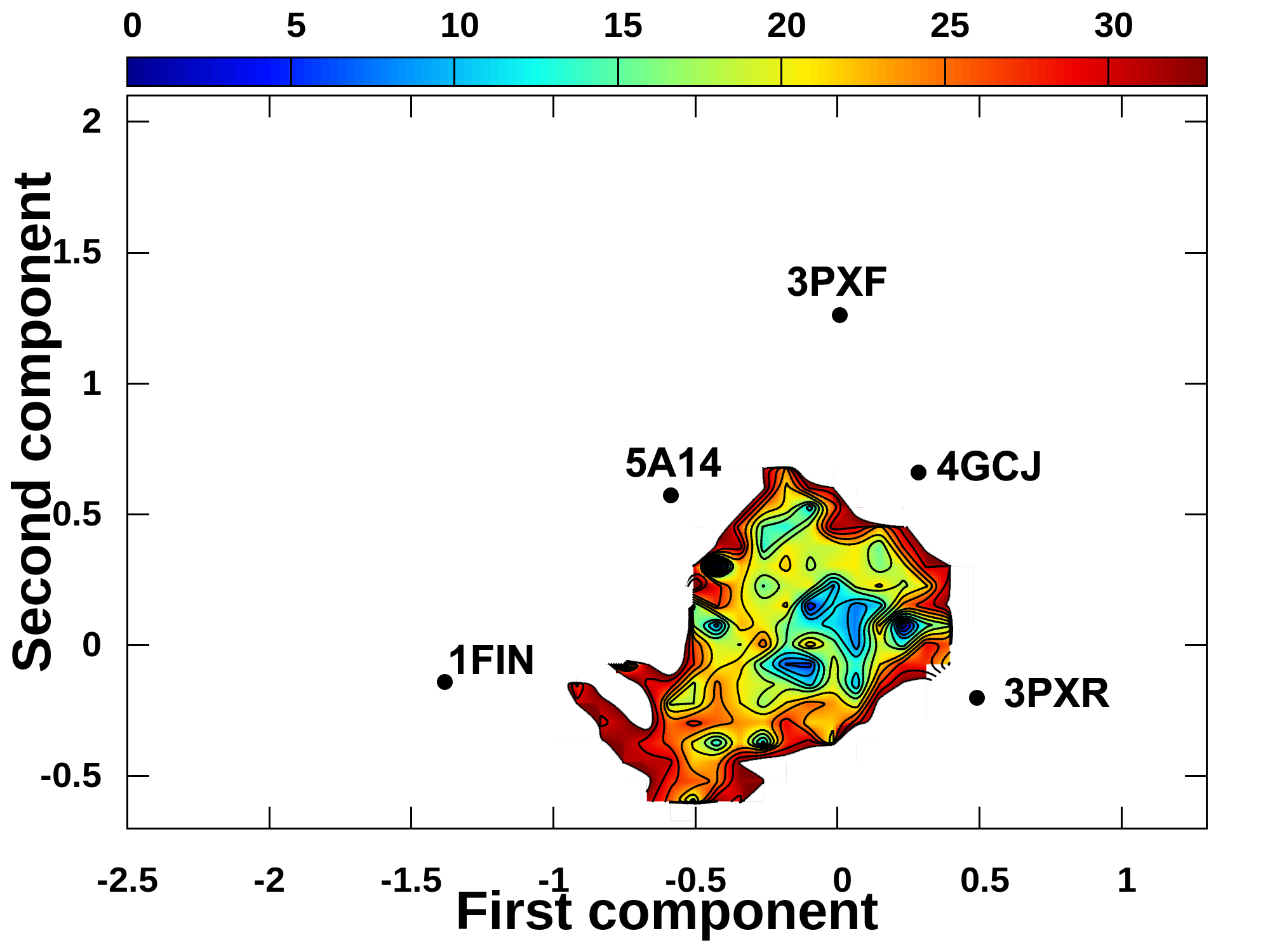

Supplement: S9 Fig — (TIF) [file pone.0154066.s009.tif]

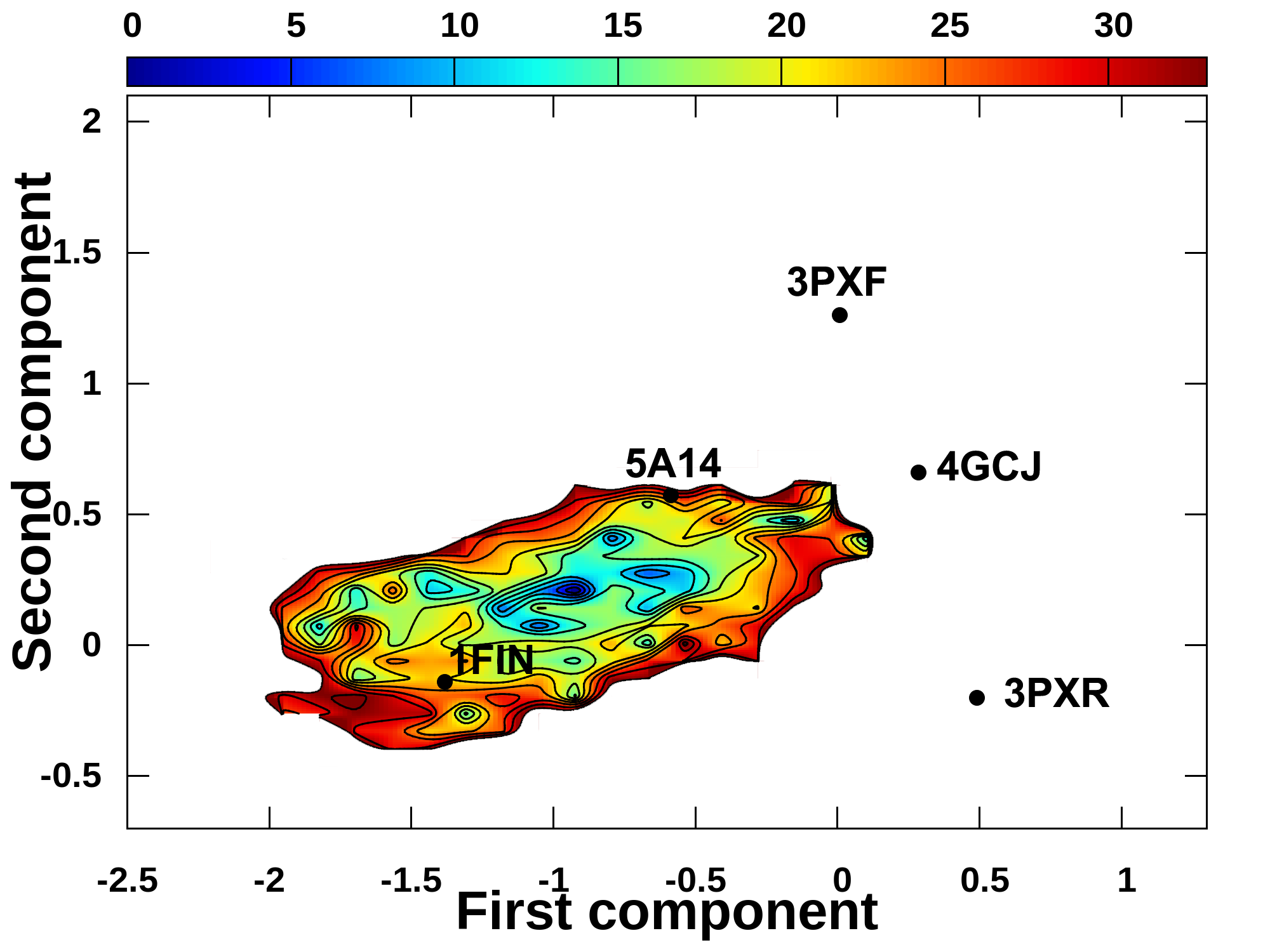

Supplement: S10 Fig — (TIF) [file pone.0154066.s010.tif]

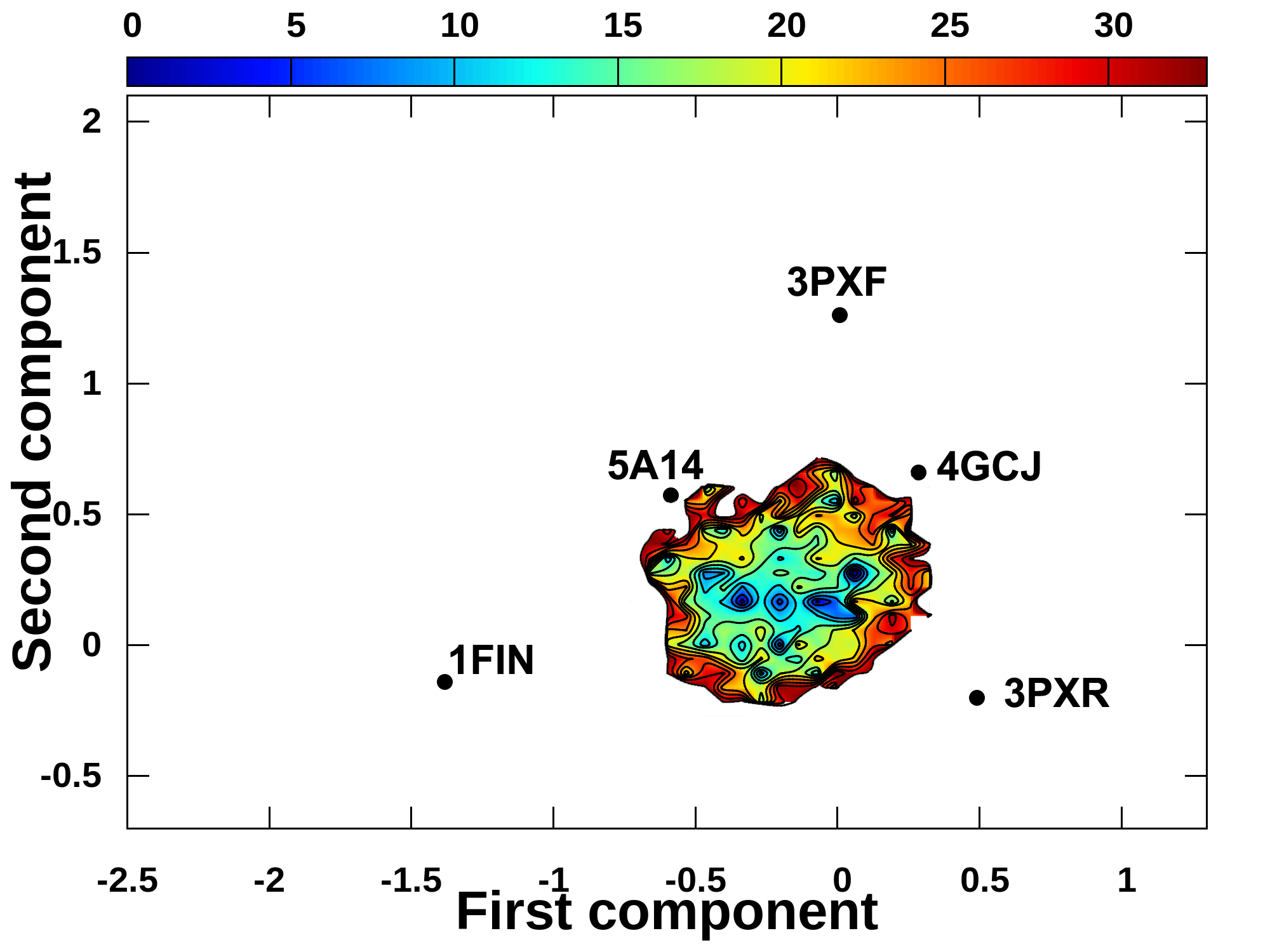

Supplement: S11 Fig — (TIF) [file pone.0154066.s011.tif]

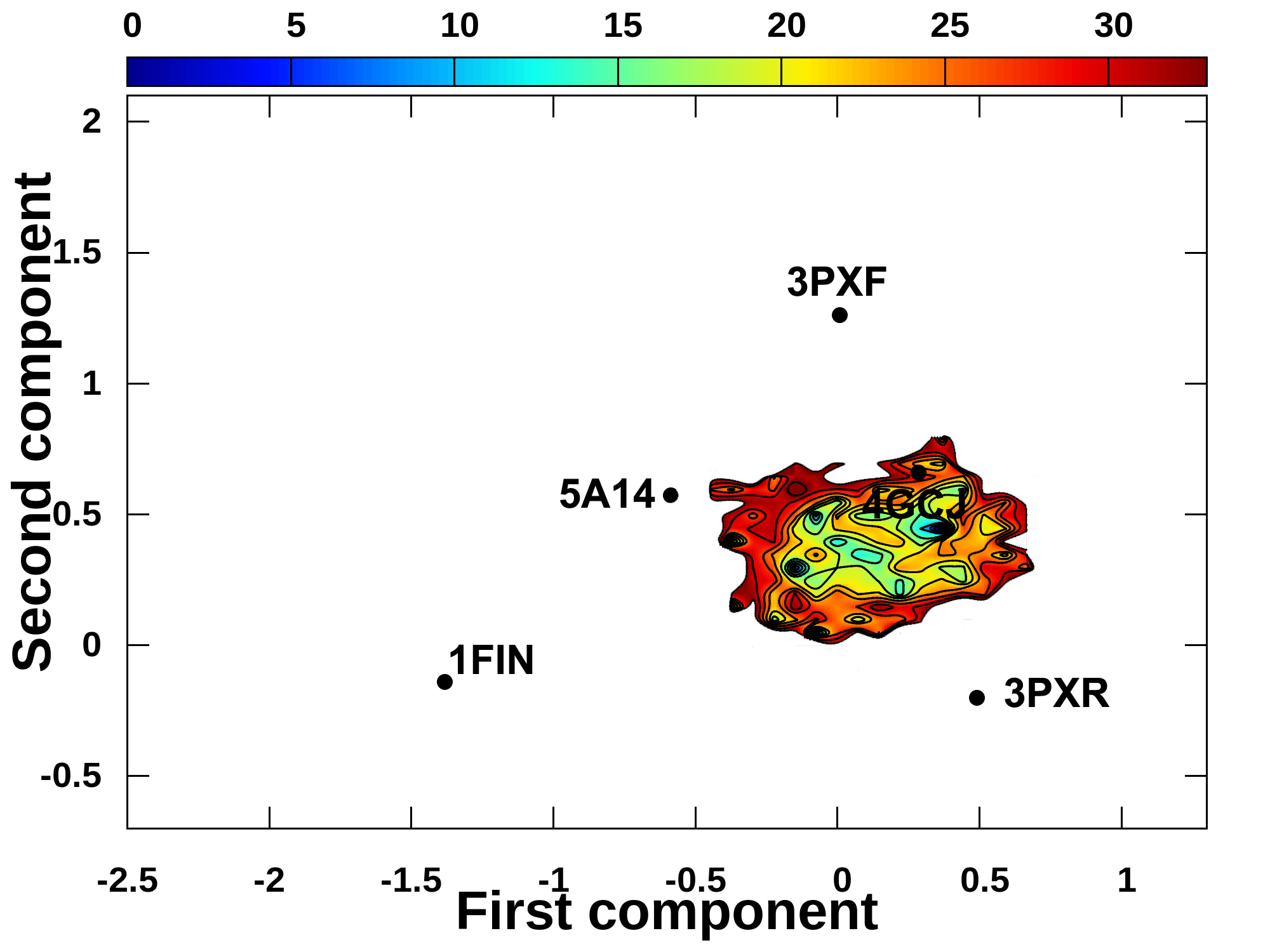

Supplement: S12 Fig — (TIF) [file pone.0154066.s012.tif]

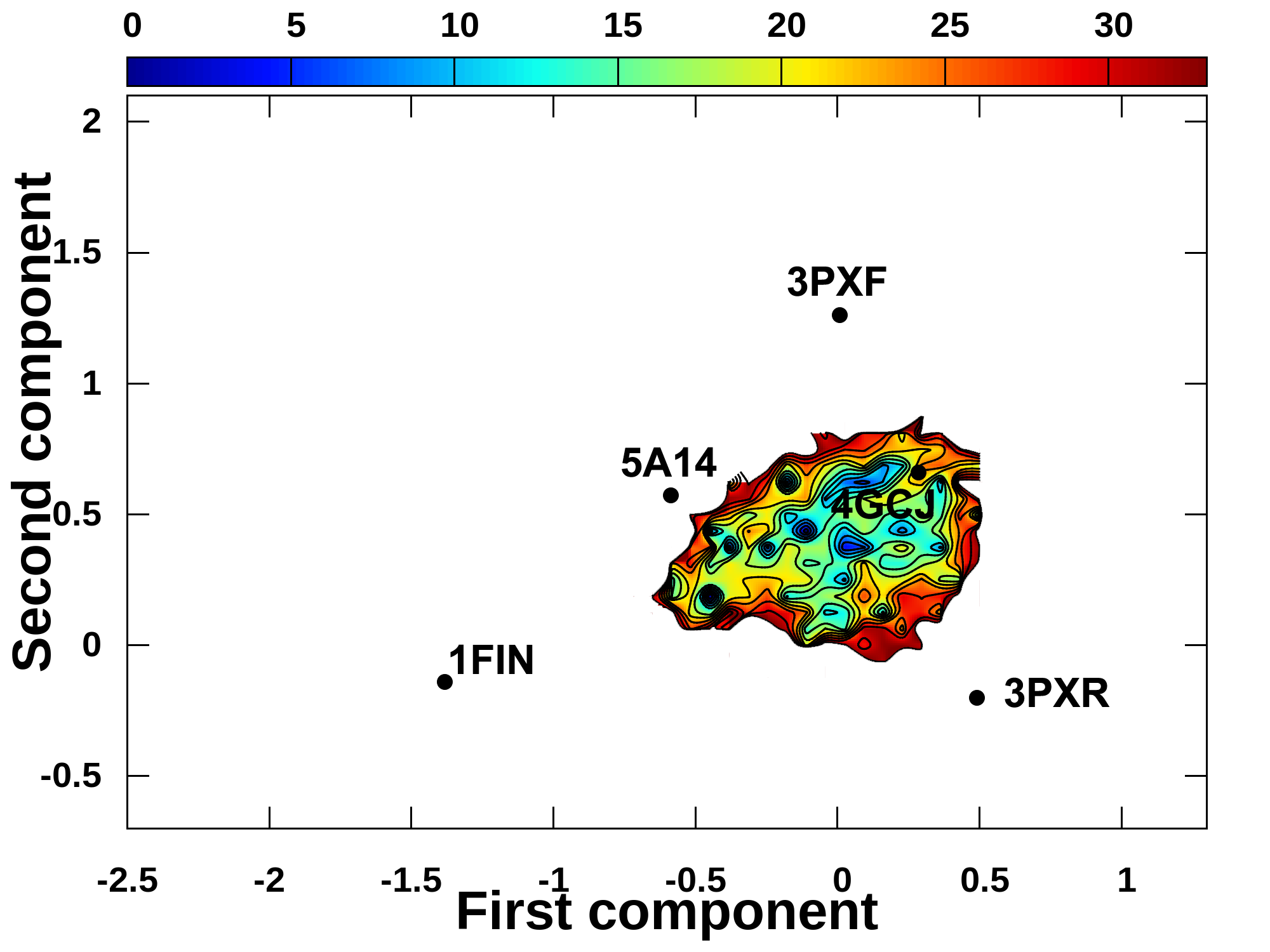

Supplement: S13 Fig — (TIF) [file pone.0154066.s013.tif]

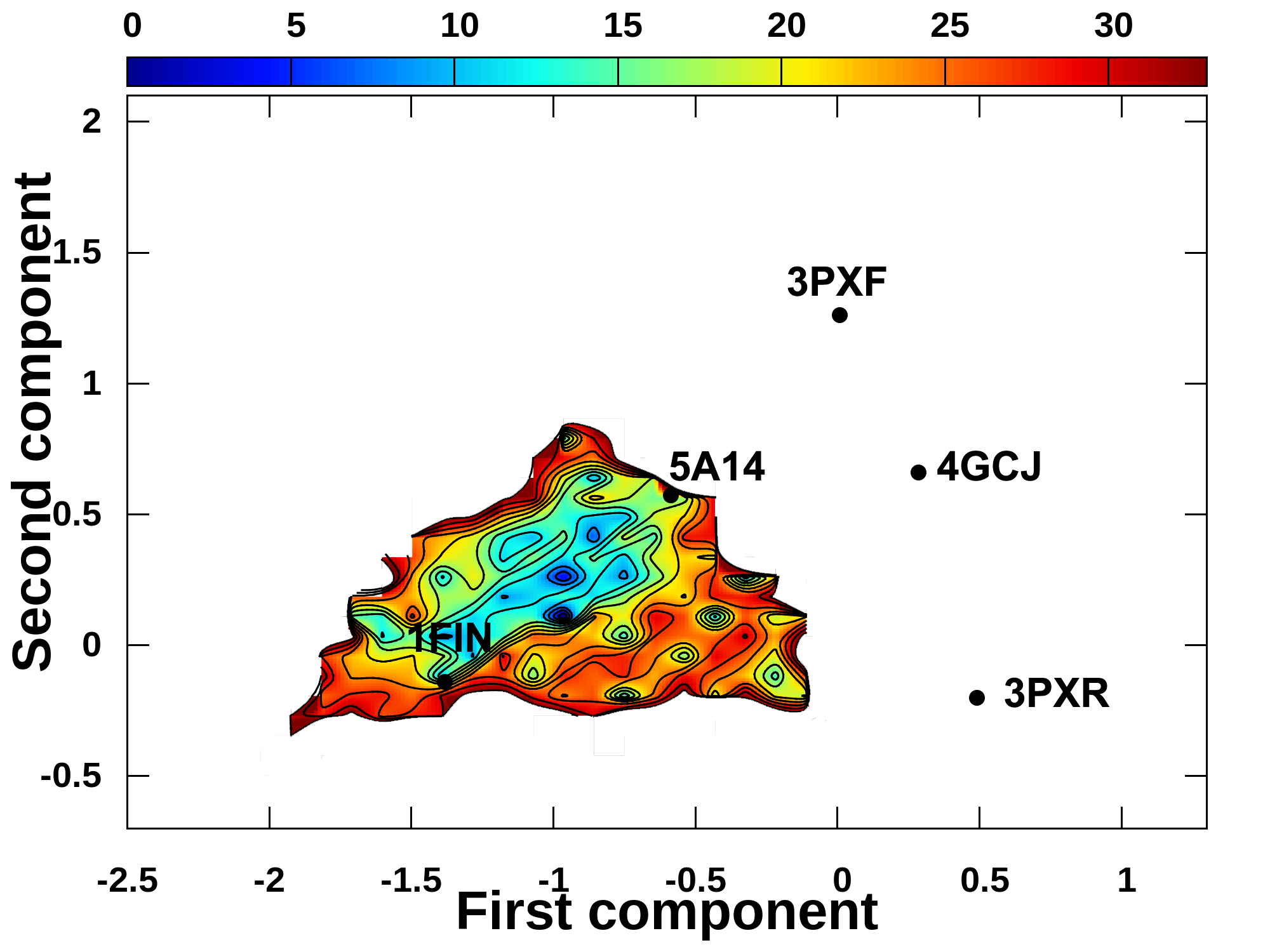

Supplement: S14 Fig — (TIF) [file pone.0154066.s014.tif]

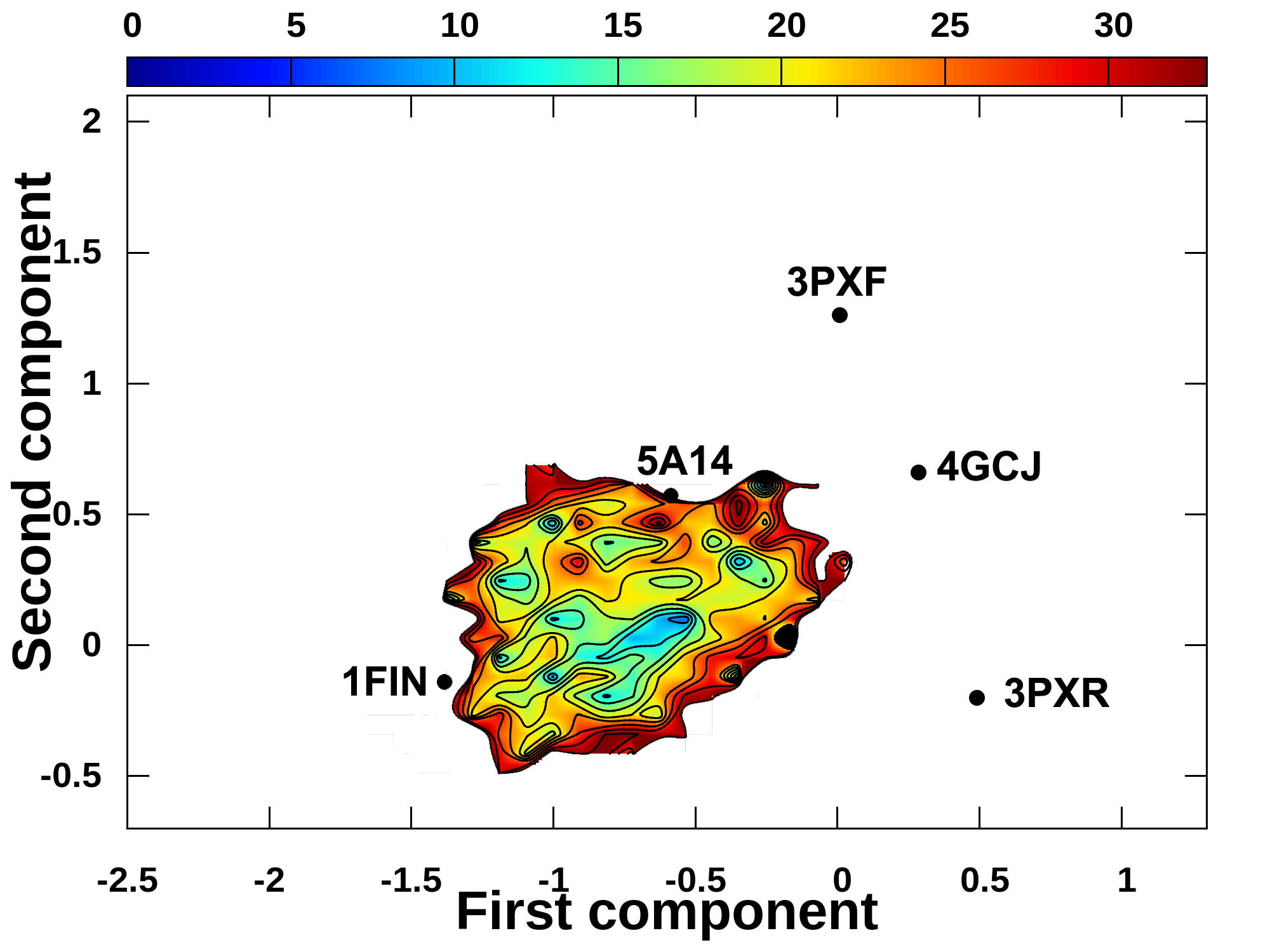

Supplement: S15 Fig — (TIF) [file pone.0154066.s015.tif]

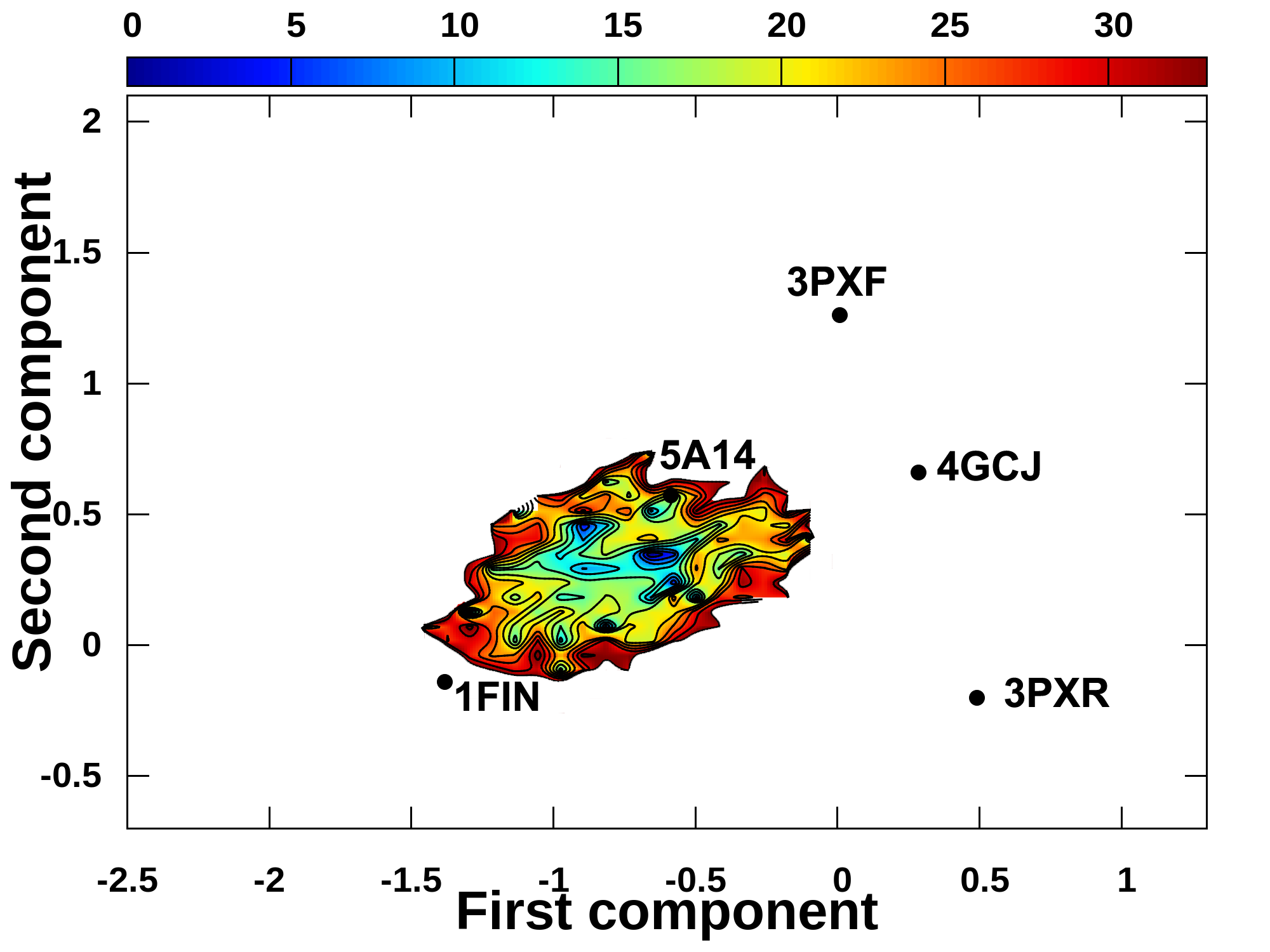

Supplement: S16 Fig — (TIF) [file pone.0154066.s016.tif]

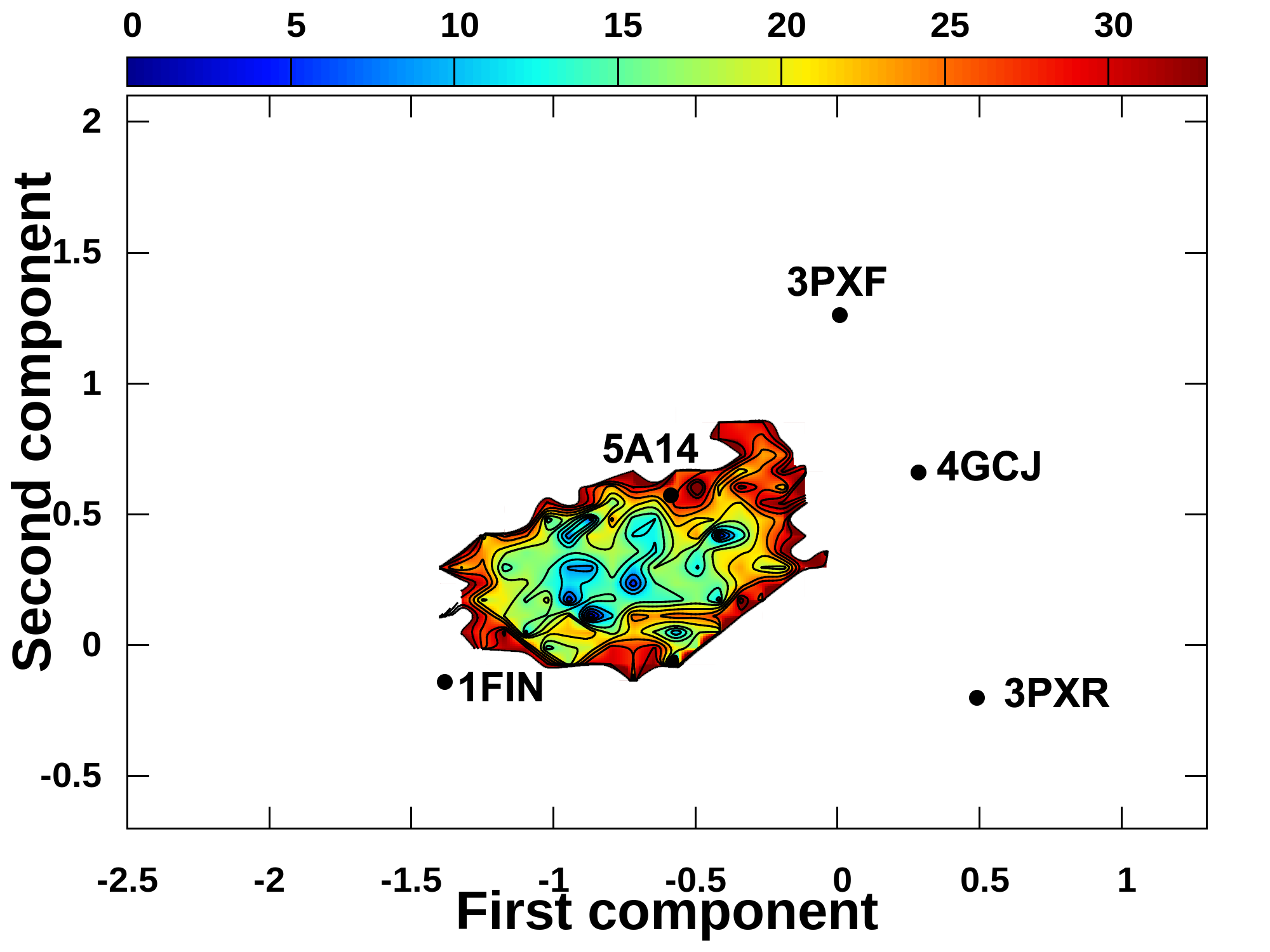

Supplement: S17 Fig — (TIF) [file pone.0154066.s017.tif]
